# Coca-Cola – a model of transparency in research partnerships? A network analysis of Coca^-^Cola’s research funding (2008–2016)

**DOI:** 10.1017/S136898001700307X

**Published:** 2018-03-21

**Authors:** Paulo M Serôdio, Martin McKee, David Stuckler

**Affiliations:** 1 Department of Sociology, University of Oxford, Manor Road Building, Manor Road, Oxford OX1 3UQ, UK; 2 Department of Health Services Research and Policy, London School of Hygiene and Tropical Medicine, London, UK; 3 University of Bocconi and Dondena Research Centre, Milan, Italy

**Keywords:** Coca-Cola, Conflict of interests, Industry-funded research, Sugar, Obesity

## Abstract

**Objective:**

To (i) evaluate the extent to which Coca-Cola’s ‘Transparency Lists’ of 218 researchers that it funds are comprehensive; (ii) map all scientific research acknowledging funding from Coca-Cola; (iii) identify those institutions, authors and research topics funded by Coca-Cola; and (iv) use Coca-Cola’s disclosure to gauge whether its funded researchers acknowledge the source of funding.

**Design:**

Using Web of Science Core Collection database, we retrieved all studies declaring receipt of direct funding from the Coca-Cola brand, published between 2008 and 2016. Using conservative eligibility criteria, we iteratively removed studies and recreated Coca-Cola’s transparency lists using our data. We used network analysis and structural topic modelling to assess the structure, organization and thematic focus of Coca-Cola’s research enterprise, and string matching to evaluate the completeness of Coca-Cola’s transparency lists.

**Results:**

Three hundred and eighty-nine articles, published in 169 different journals, and authored by 907 researchers, cite funding from The Coca-Cola Company. Of these, Coca-Cola acknowledges funding forty-two authors (<5 %). We observed that the funded research focuses mostly on nutrition and emphasizes the importance of physical activity and the concept of ‘energy balance’.

**Conclusions:**

The Coca-Cola Company appears to have failed to declare a comprehensive list of its research activities. Further, several funded authors appear to have failed to declare receipt of funding. Most of Coca-Cola’s research support is directed towards physical activity and disregards the role of diet in obesity. Despite initiatives for greater transparency of research funding, the full scale of Coca-Cola’s involvement is still not known.

There is longstanding concern that multinational companies manufacturing products harmful to health fund research seeking to prevent public health policies designed to counter the effects of their products. Thanks to the disclosure of tobacco industry documents, much has been learnt about how that particular industry conducted research designed to create confusion and to reframe the agenda in ways that advanced its interests^(^ ^1^ ^–^ ^4^ ^)^. Recently attention has turned to similar activities by the food industry. In early 2015, Coca-Cola attracted extensive criticism when it was revealed that it had funded a ‘Global Energy Balance Network’ (GEBN), led by John Peters and James Hill (University of Colorado), Gregory Hand (West Virginia University) and Steven Blair (University of South Carolina), whose main message was that there was no compelling evidence of a significant link between sugar-sweetened beverages and obesity^(^ ^5^ ^)^. The funding agreement with the GEBN was not visible for public scrutiny, as none of the parties involved had disclosed it on their websites, and it was made available by the recipient universities only in response to requests under freedom of information laws. However, the scale and influence of hidden research by the food and beverage industry are unclear and, so far, there has been a dearth of research on the role of vested interests such as Coca-Cola’s.

One methodological challenge is identifying those articles funded by specific actors. Previously, search tools such as Web of Science, PubMed or MEDLINE have not facilitated this. However, in 2008 Thomson Reuters implemented a large-scale indexation of the paratextual information on funding acknowledgement statements and made them available for searching in one of its databases, the Web of Science Core Collection. Building on this innovation, we developed an algorithm in R programming language that crawls the results of a Web of Science search on funding statements and scrapes, parses and compiles the metadata from the studies identified by the search, so making it possible to review systematically research funded by Coca-Cola.

Using this new dimension of bibliometric analysis and the innovative tool we designed, we undertook a systematic review of the extent of involvement of Coca-Cola in funding nutrition research. We further took advantage of a unique opportunity to evaluate the extent to which Coca-Cola is transparent and comprehensive in its disclosures.

In September 2015, Coca-Cola published a ‘Transparency List’ of 115 ‘Health Professionals and Scientific Experts’ and forty-three ‘Research Projects’ that it sponsored in the USA^(^ ^6^ ^,^ ^7^ ^)^. Following this disclosure, some of Coca-Cola’s subsidiaries and bottlers published similar transparency lists for health and wellness partnerships and financial support of scientific research in the UK^(^ ^8^ ^)^, France^(^ ^9^ ^)^ and Germany^(^ ^10^ ^)^, in December of 2015, and in Australia^(^ ^11^ ^)^, New Zealand^(^ ^12^ ^)^ and Spain^(^ ^13^ ^)^, in March of 2016 (see online supplementary material 1 for the full lists of health professionals and scientific experts).

Here, using this new instrument, we investigate the following questions:

1. Are Coca-Cola’s transparency lists complete?
2. How many studies and authors are funded by the Coca-Cola brand?
3. Which research topics and interventions are supported by Coca-Cola funding?
4. Are Coca-Cola funded researchers declaring their links to the company in their publications?

## Materials and methods

### Data

Following PRISMA (Preferred Reporting Items for Systematic Reviews and Meta-Analyses) guidelines, we reviewed research supported by Coca-Cola funding using the Web of Science Core Collection database^(^ ^14^ ^)^. Starting in 2008 Thomson Reuters added funding acknowledgement and competing interest statements to all the bibliographic records of the Science Citation Index Expanded. This retrieves the funding/competing interest paratextual information in the published version of an article as well as distinguishing between a conflict of interest and a funding statement, and identifies the entities that are acknowledged as providing funding for the article – saving the user from having to read the statements and identify the funding sources manually. These changes, in contrast to other existing databases, now enable users to search the database for text strings (e.g. names of corporations) in the funding acknowledgement section, either as a funding agency or simply as part of a declared conflict of interest.

To our knowledge, Web of Science is the only bibliographic database to index this information on a large scale (Scopus developed a similar algorithm, but with a considerably lower coverage of publications and for a shorter time period; and, more recently, PubMed started adding this information to the metadata of the publication records it indexes).

To retrieve metadata from the literature searched, we developed a web scraping tool that crawls the URL address of any search run in the Core Collection database of Web of Science. Our algorithm, written for R software, runs sequentially over each study page in the search results, parses the HTML code and scrapes user-defined fields for each publication (e.g. title, abstract, authors and affiliated institutions), including the funding/competing interest statement and a table compiled by Web of Science that lists all the entities that provided funding for the article, as reported by the authors (the R script for the algorithm is provided in online supplementary material 2).

**Fig. 1:**
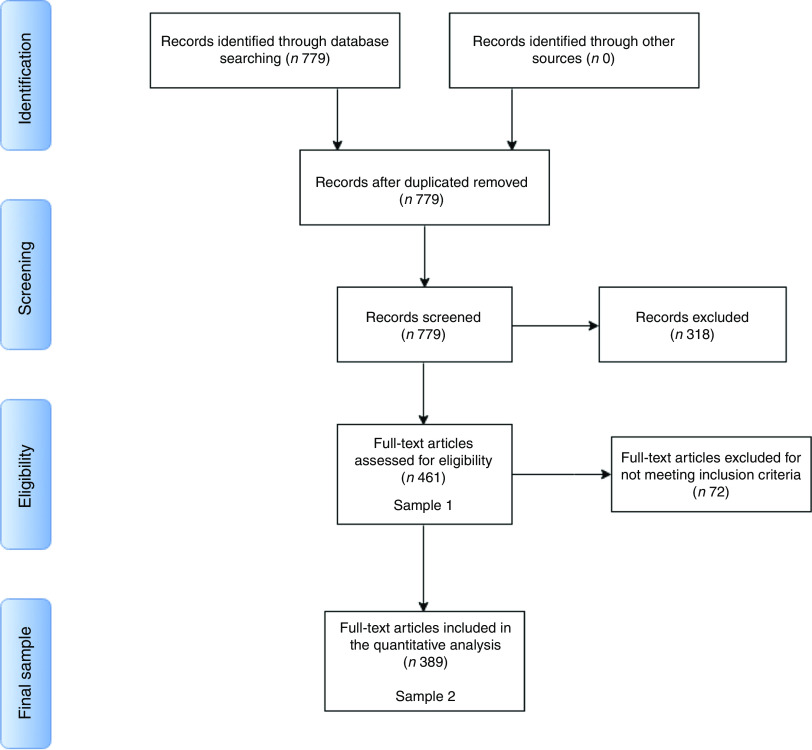
PRISMA (Preferred Reporting Items for Systematic Reviews and Meta-Analyses) flow diagram for the present systematic review. This PRISMA diagram describes the study selection steps and identifies at what stage we arrived at analytical Samples 1 and 2. Seven hundred and seventy-nine records (i.e. publications) were retrieved from a search on the ‘funding text’ field in Web of Science for any mention of ‘Cola’. From these records, 318 were excluded for not meeting the screening criteria (i.e. the study acknowledging direct receipt of funding from Coca-Cola). After exclusion, we arrive at Sample 1, which contains all studies funded by the Coca-Cola brand. We subset from Sample 1 only those studies funded by The Coca-Cola Company, its affiliates in the USA and those subsidiaries that published transparency lists; this leads to the exclusion of seventy-two studies for not meeting the eligibility criteria, which gives us Sample 2

We searched for all studies that included the string ‘cola’ in the ‘funding text’ field, which indexes the entire funding acknowledgement section as reported in the published manuscript (see Fig. 1 and Appendix 1 for search strategy). This broad search strategy identified 779 articles, published between 2008 and June 2016, and included articles that acknowledged both direct funding and competing interests involving The Coca-Cola Company and all its subsidiaries. In addition, the broad search term ‘cola’ also yielded studies funded by other companies, such as ‘Pepsi-Cola’.

The questions set out above make an implicit separation between the research funding activities of The Coca-Cola Company and those of the Coca-Cola brand, which includes all subsidiaries and bottlers around the world. With this distinction in mind, we constructed two analytical samples.

The first sample is less restrictive, composed of all studies directly funded by any company or institute part of the Coca-Cola brand that were retrieved from our search. This sample is used to answer question 2 (‘How many studies and authors are funded by the Coca-Cola brand?’).

The second sample is a sub-sample of the first, focusing on those studies funded by The Coca-Cola Company and its philanthropic arms in North America, and by the subsidiaries and bottlers that participated in the ‘Transparency Initiative’. This sample is used to answer the remaining questions (1, 3 and 4).

Below, we describe all steps in the selection of the studies and in the creation of the two analytical samples.

### Study selection

The 779 studies produced by our search were screened for the inclusion of the string ‘Coca-Cola’ (or any possible variant, including affiliates of the main company, such as the Beverage Institute of Health and Wellness^(^ ^15^ ^)^; see Appendix 1 for a list of all variants) as a funding agency. The goal of the initial screening was to parse through the search results and only keep studies that report direct receipt of funding from The Coca-Cola Company or any affiliates (see Fig. 1).

This criterion thus excluded 318 studies. These were studies where: (i) the authors only declare a competing interest due to previous relationships with The Coca-Cola Company unrelated to research funding (e.g. speaking engagements or consultancy work); (ii) Coca-Cola’s involvement in the publication was indirect (e.g. via student grants); (iii) the authors acknowledge funding from another ‘cola’, such as ‘Pepsi-Cola’; and (iv) where the algorithm used by Web of Science mistakenly included Coca-Cola as a funding agency, when the funding acknowledgement section did not indicate direct funding by the company to that particular study (this was assessed by manually inspecting all funding statements).

To be eligible for our first sample, studies had to acknowledge funding from The Coca-Cola Company or any of its affiliates, including The Coca-Cola Foundation (TCCF, the philanthropic arm of the company), Coca-Cola North America, The Beverage Institute for Health and Wellness (an organization set up by The Coca-Cola Company to support nutrition research)^(^ ^15^ ^)^ and Coca-Cola bottlers or subsidiaries outside the USA. This criterion comprises the totality of the Coca-Cola brand in our data and did not lead to the removal of any further studies. Sample 1 is therefore comprised of 461 studies.

In the second sample, we imposed stricter eligibility criteria to isolate those studies funded by The Coca-Cola Company and its affiliates in the USA, France, Germany, Spain, New Zealand and Australia, the only countries to release records of their research funding efforts in the form of transparency lists of funded scientific experts, which were released in late 2015 and early 2016 (see full lists in online supplementary material 1).

This criterion excluded seventy-two studies that were funded by subsidiaries or bottlers other than the ones listed above. Sample 2 is thus comprised of 389 studies.

### Analysis

To answer the first question, concerning how comprehensive was Coca-Cola’s transparency initiative, following the revelation of its financial backing of the GEBN, we recreated Coca-Cola’s lists of ‘scientific experts’ and ‘research partnerships’ by carefully following the parameters laid out in Coca-Cola’s transparency disclosure^(^ ^6^ ^,^ ^7^ ^)^, using our own data on funding statements retrieved from Web of Science (Sample 1). We then matched our recreated list to the original ones published on Coca-Cola’s websites^(^ ^6^ ^–^ ^13^ ^)^. This was designed to identify any discrepancy that could, potentially, reflect selective disclosure on the Company’s part.

Coca-Cola included in its ‘Research and Partnerships’ lists the names of academics it funded or collaborated with according to the following criteria (these can be found on the company’s websites)^(^ ^6^ ^–^ ^13^ ^)^: (i) funding agreements sourced exclusively from The Coca-Cola Company, The Coca-Cola Foundation, Coca-Cola North America, Coca-Cola South Pacific, Coca-Cola Australia Foundation, Coca-Cola Oceania, Coca-Cola Germany and Coca-Cola Spain; and (ii) activities and studies conducted between January 2010 and December 2015.

To match these criteria, we started with the 461 studies in Sample 1 and excluded the following: (i) studies published before 2010 and after December 2015; (ii) studies funded by Coca-Cola subsidiaries and bottlers, with the exception of those listed above; (iii) studies written as part of research consortia that were themselves funded by The Coca-Cola Company, since the funding link between the company and the publication is indirect (see online supplementary material 1, Supplemental Table 1 for a complete listing of such consortia); and (iv) authors who were not listed as principal or co-investigators on the Coca-Cola grant in the original funding statement, where this information was made available (unfortunately, most funding statements did not identify the main investigator on the grant). We opted for a conservative method of removing studies to guarantee, to the highest degree possible, an approximation to the way Coca-Cola compiled its own lists of funded researchers.

One hundred and thirty-eight studies did not meet the eligibility criteria and were removed from the matching procedure.

It should be noted that although there is a gap between the time funding is awarded and the publication date of a study, which suggests that we should restrict our parameters to publications from 2012 onwards, it is not clear from the information provided by Coca-Cola that authors of research published in 2010 would not be included in its transparency list. In fact, it is the case that some studies yielding publications in 2010 were still ongoing in subsequent years. Furthermore, a large proportion of authors who published in 2010 also appeared in published research later on, which suggests that projects funded by Coca-Cola were likely to have yielded more than one publication over time. Therefore, the method we designed to match our data to Coca-Cola’s lists includes research published from 2010 onwards.

Notwithstanding, to confirm the validity of our method, we used a sub-sample of studies published between 2012 and 2015 to compare with Coca-Cola’s transparency lists; the results lend further support to our findings using studies published from 2010 onwards (see ‘Limitations of the study’ section below).

After exclusion of ineligible studies, the procedure identified 907 authors, responsible for 331 studies that fit the criteria used by Coca-Cola to compile its lists of funded research partnerships. The combined transparency lists published by Coca-Cola in the USA, UK, Australia, France and Germany (Spain and New Zealand did not contain names of individual researchers) named 218 researchers. We then proceeded with matching the names of the 907 authors we identified in our data to the 218 names of researchers listed by Coca-Cola as recipients of its research funding, using whole and approximate string matching with manual verification of the results.

**Fig. 2:**
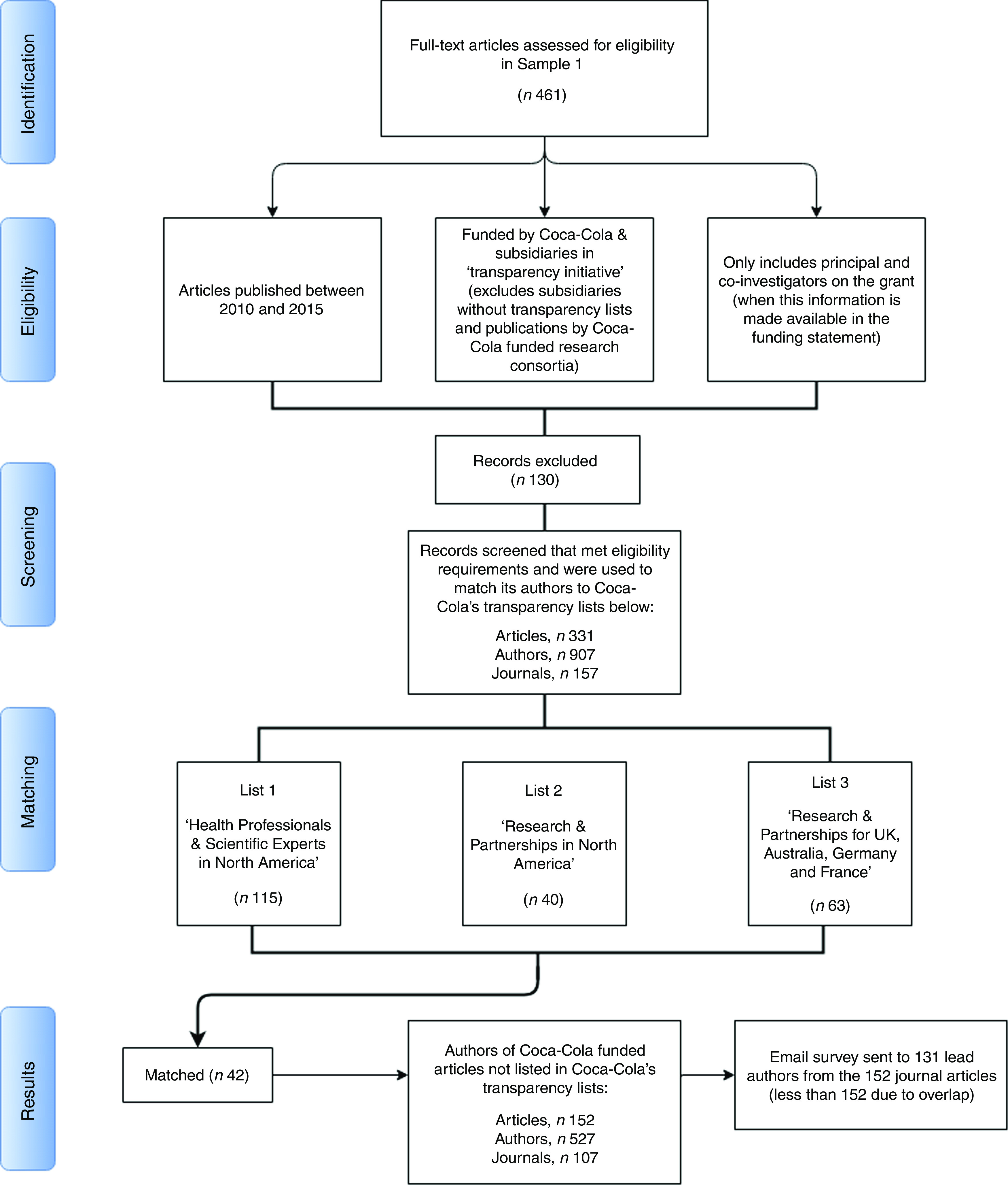
Flow diagram of the process to match Web of Science data to Coca-Cola’s transparency lists. This flow-type diagram describes: (i) the steps taken to recreate Coca-Cola’s transparency lists using our data; and (ii) the matching of our recreated list to Coca-Cola’s combined transparency lists. We start with all studies in Sample 1 and begin evaluating them against the parameters that governed Coca-Cola’s lists of scientific experts and researchers it funded, and excluding those that failed to meet the eligibility criteria. In the matching stage, we combined the lists of researchers funded by Coca-Cola in North America, UK, Australia, Germany and France and matched these names to those on the list we created using data from Web of Science. The corresponding author on studies with all unmatched names were surveyed via email and asked about Coca-Cola funding

Figure 2 summarizes this iterative method in a PRISMA-type diagram.

The second question raised above seeks to reveal the universe of scientific literature funded by Coca-Cola. For this question, we focused on the Coca-Cola brand as a whole, not making any distinction between the research funding activities carried out by the main company in the USA and those of its subsidiaries and bottlers around the world.

To address this question, we employed network analysis tools to visually portray the scope of Coca-Cola’s involvement in funding scientific research, and at the same time compare it with the company’s disclosure following its transparency initiative. We built co-authorship networks for all studies that were funded by the Coca-Cola brand between 2008 and 2016. The diagrams show nodes (authors) linked via edges, which represent the co-authorship of a study. A similar approach has been used in the literature combining a systematic review with co-citation networks, instead of co-authorship networks^(^ ^16^ ^,^ ^17^ ^)^.

Network analysis was paired with text analysis to assess the content of the scientific literature funded by Coca-Cola. In addressing question 3, we shift our focus to the funding endeavours of the Coca-Cola Company and those affiliates that participated in the transparency initiative, and discuss who and what fields of research they funded between 2008 and 2016. We added a new co-authorship network and ran a community search algorithm^(^ ^18^ ^)^ to uncover highly cohesive subgroups that may indicate the presence of different research hubs throughout the USA (and abroad).

The algorithm calculates betweenness centrality scores for each tie in the network, a metric that counts the number of shortest paths between all pairs of nodes that pass through each tie. In short, it counts how often a tie is used as a ‘bridge’ to connect, in the shortest way possible, any two pair of nodes. It proceeds by removing the tie with the highest score of betweenness, recalculating tie betweenness centrality and iteratively removing ties with the highest betweenness score until the network becomes disconnected into several subgroups. Once it achieves an optimal number of subgroups, the partitioning of the network is complete and it assigns different colours to each subgroup.

This methodology offers valuable insights on the structure and organization of Coca-Cola’s research enterprise, as it furthers our understanding of its centralization, which actors are important and whether research themes or institutions may play a role in its organization. Furthermore, it puts Coca-Cola’s transparency initiative in perspective, both in terms of scope (how complete is the disclosure) and in terms of relevance (whether the authors the company acknowledge as recipients of funding are central or peripheral players in the network).

To better understand the research themes of Coca-Cola’s funded research (the second part of question 3), we examined the abstracts of all 389 articles that met the screening and eligibility criteria that underpinned Sample 2. Using structural topic modelling^(^ ^19^ ^)^, a variant of the large toolbox of topic modelling estimation methods, generally described as unsupervised machine learning algorithms for probabilistic classification of large text corpora, we uncovered hidden semantic structures, or topics, that give us an insight into the different streams of research that Coca-Cola has funded since 2008.

In a nutshell, topic models estimate latent topics in a bundle of text documents and simultaneously assign the documents to the different topics, probabilistically. The algorithm works on the assumption that a document is composed of a different mixture of topics and estimates the probability distribution of documents to topics; it does this based on the semantic content of each document by leveraging information on the word frequency within and across documents. Thus, documents that share the same semantic structure (i.e. similar distributions of word frequencies) are likely to belong to the same topic.

In online supplementary material 3 we present in greater detail the estimation methods and robustness tests for the models presented here.

In the next section, the results are organized and discussed around each of the research questions set out above.

## Results

### Testing the completeness of Coca-Cola’s transparency lists

Our search in Web of Science identified a total of 907 authors corresponding to 331 studies. These were the studies that met the eligibility criteria required to match their authors onto Coca-Cola’s transparency lists (for a full list of the studies included in Sample 1, Sample 2 and in the sample used to match against Coca-Cola’s transparency lists, see online supplementary material 1, Supplemental Table 2).

To evaluate the degree of transparency, first we compared the 907 names with the 218 researchers and scientific experts named directly by the company and selected subsidiaries as recipients of its own research funding. Forty-two people appear in both sets (see Fig. 2). This corresponds to 20 % of the names on Coca-Cola’s transparency lists and to 4 % of the names listed in Web of Science as authors of Coca-Cola funded publications.

Next we performed a series of robustness checks and tests for alternative possibilities.

First, as only one researcher per publication could be the direct recipient of a grant, we removed all publications involving any of the forty-two authors whom we identified successfully. There still remained 527 authors corresponding to 152 published articles that acknowledge Coca-Cola funding but were not named on Coca-Cola’s transparency lists.

Second, we surveyed via email 131 (fewer than 152 because of overlap) corresponding authors requesting whether they had received funding from Coca-Cola or not during the period 2010 to 2015, using the corresponding email address indicated in the manuscript. Each corresponding author was emailed twice over the course of 20 d. It should be noted that the corresponding author is, in many cases, a junior researcher on a publication, thus unlikely to be the principal investigator on the grant. Eleven per cent (fourteen authors) confirmed Coca-Cola funding, 22 % (twenty-nine authors) denied it and 53 % (sixty-eight authors) did not reply. In cases where the respondent denied funding, we asked who were the primary recipient(s) of the grant. The remaining 14 % of email addresses were no longer valid.

We altered our sample according to the results of the survey in the following manner: (i) in the cases where the respondent denied funding, we removed the respondent’s name from the sample and, in the few cases where the respondent provided the name of the primary recipient(s) of the grant, we kept the latter’s name in the sample; and (ii) in cases where the respondent confirmed receipt of funding, we removed the names of all co-authors on each publication of the respondent in the sample.

After incorporating the results from the survey, our search identified up to 471 authors corresponding to 128 articles whose names do not appear on Coca-Cola’s lists, but whose articles acknowledge funding from the company.

### Mapping the universe of Coca-Cola’s research funding: a network analysis

Next, we did a broad search for all published research acknowledging financial support from any member of the Coca-Cola brand (including the main company, subsidiaries, bottlers and other affiliates), for the entire period for which we have data, 2008 to 2016.

**Fig. 3:**
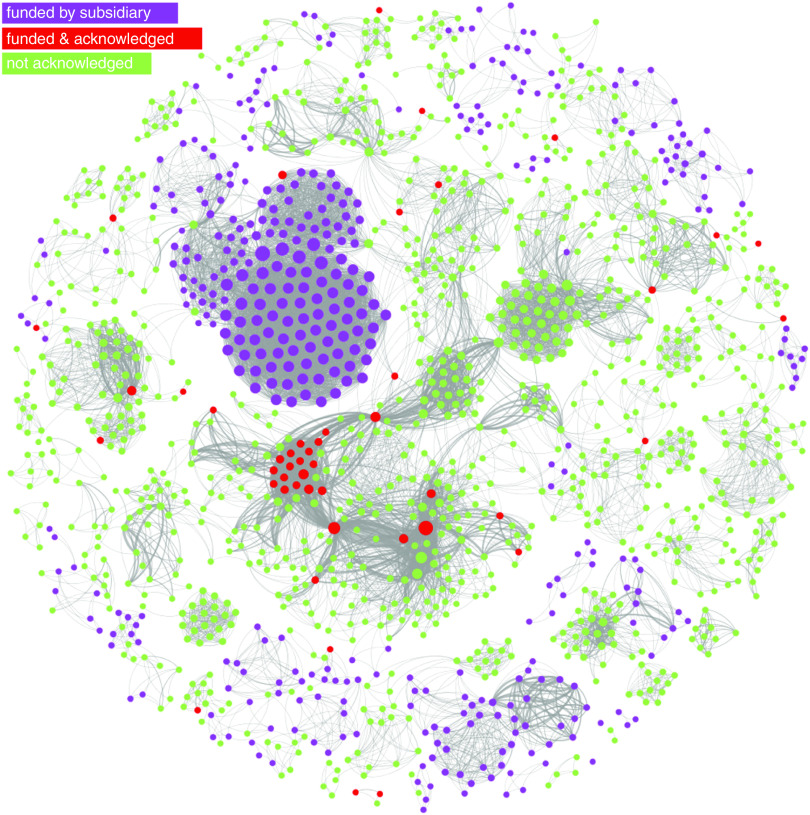
Network of linkages between authors of publications acknowledging Coca-Cola related funding. This network graph shows co-authored publications (ties) between authors (nodes), for publications that acknowledge funding from The Coca-Cola Company, The Coca-Cola Foundation, the Beverage Institute for Health and Wellness and any subsidiary or bottler company (e.g. Coca-Cola Brasil). Nodes in red identify authors who appear on Coca-Cola’s transparency lists. Nodes in green identify authors on Coca-Cola funded publications whose names do not appear in Coca-Cola transparency lists. Nodes in purple identify authors on publications funded by Coca-Cola subsidiaries, also not on Coca-Cola’s lists. Nodes are sized by degree centrality (total number of co-authors times the number of shared publications they have)

Figure 3 depicts a network of co-authorships, where nodes represent authors and ties represent shared publications between them, for all publications that acknowledge funding from the Coca-Cola brand. Thicker lines denote a higher number of co-authored publications between any two nodes. Nodes were sized by degree centrality, a network measure that captures how central is a node in the network by adding up the weights of its ties (in this case, it will reflect the total number of co-authored publications for each author).

The colour partitioning of the network allows us to compare Coca-Cola’s disclosure with the known universe of Coca-Cola funded research since 2008. Nodes marked in red represent the forty-two authors whom we were able to match to Coca-Cola’s transparency lists. Nodes in green represent authors on studies (from Sample 2) that declared funding from The Coca-Cola Company, Coca-Cola North America, Beverage Institute for Health and Wellness and The Coca-Cola Foundation, but who were not acknowledged in the company’s transparency list. Finally, we extend the network to the whole brand (Sample 1) by colouring in purple nodes representing authors of studies funded by Coca-Cola subsidiaries, bottlers or affiliate companies that share the brand name around the world, such as Coca-Cola Brasil and Coca-Cola Hellas, and who were equally absent from those subsidiaries’ transparency lists.

The network analysis reveals that the researchers acknowledged by Coca-Cola, albeit occupying a central position in the graph, represent only a small subset of the universe of research reporting Coca-Cola funding, which involves 1496 different researchers (we assume not all grant recipients) and 12 412 co-authorship ties, corresponding to 461 publications funded by the brand. The network is sufficiently disconnected for us to find several self-contained cliques of researchers, detached from the main component of the graph, and whose names did not feature in any of Coca-Cola’s lists. In other words, Coca-Cola’s transparency list appears to cover only a small portion of research in which the company is involved.

Additionally, the network structure shows several highly dense and autonomous research groups, disconnected from the main component of the network, and with no ties to the researchers acknowledged by the company. In addition, there are other equally central nodes in the network that were not acknowledged by Coca-Cola. This suggests the company is funding several research groups but has acknowledged only a subset.

However, a caveat to this visual assessment of the completeness of Coca-Cola’s lists is that it includes a small portion of publications that precede the start date of these lists (2010) and publications funded by subsidiaries that did not participate in the transparency initiative of the main company.

### Who and what is The Coca-Cola Company funding? A topical analysis of abstracts

Now we turn to mapping who and what Coca-Cola is funding, seeking to understand what areas of research and who are the academics getting its financial support.

Table 1 reports the most prolific Coca-Cola funded authors (see online supplementary material 1, Supplemental Table 3 for the top fifteen institutional affiliations; the metadata for the studies included in these two tables are available upon request). As shown in Table 1, the researcher who has published the most articles with Coca-Cola funding is a former president of the American College of Sports Medicine (S.B.). He has received around $US 5·4 million of research funding to study the role of energy balance at high levels of energy intake^(^ ^7^ ^)^, and he also played a pivotal role in the creation of the GEBN^(^ ^5^ ^)^ (see online supplementary material 1, Supplemental Table 4).

**Table 1:** Top fifteen most frequent authors in Sample 2

Rank	Researcher’s name	No. of articles	On Coca-Cola’s transparency list?
1	Steven Blair	89	Yes
2	Xuemei Sui	54	No
3	Timothy Church	51	Yes
4	Duck-Chul Lee	39	No
5	Peter Katzmarzyk	27	Yes
6	Carl Lavie	24	Yes
7	Gregory Hand	22	Yes
8	James Hébert	17	No
9	Mark Tremblay	17	Yes
10	Conrad Earnest	16	No
11	Steven P Hooker	15	No
12	William Koros	15	No
13	Olga Sarmiento	15	Yes
14	Robin Shook	15	No
15	Jean-Philippe Chaput	14	Yes

Sample 2 includes only those studies funded by The Coca-Cola Company and its affiliates in the USA, France, Germany, Spain, New Zealand and Australia, the only countries to release records of their research funding efforts in the form of ‘Transparency Lists’ of funded scientific experts, which were released in late 2015 and early 2016 (see full lists in online supplementary material 1). Sample 2 was compiled by the authors using data retrieved from the Web of Science Core Collection. The metadata for the studies included in this table are available upon request.

Other leading Coca-Cola researchers are: a former Dean of the School of Public Health of West Virginia University (G.H.), who was the Principal Investigator on ‘Energy Flux – are we healthier when energy balance is achieved’ (funded with $US 851 000 by Coca-Cola), which resulted in the Energy Balance Study^(^ ^20^ ^)^, designed to evaluate the impact of energy intake and expenditure on changes in weight; and a member of the American Society for Nutrition and the Canadian Diabetes* who received two unrestricted grants ($US 192 000) in 2014 from Coca-Cola and has argued that there is no convincing evidence that added sugars in the diet have a unique impact on the development of obesity or diabetes^(^ ^21^ ^)^ (J.S.). His research showing there is no association between total sugars intake and risk of diabetes^(^ ^22^ ^,^ ^23^ ^)^ has informed the Canadian Diabetes Association’s position statements on sugars^(^ ^24^ ^)^.

**Fig. 4:**
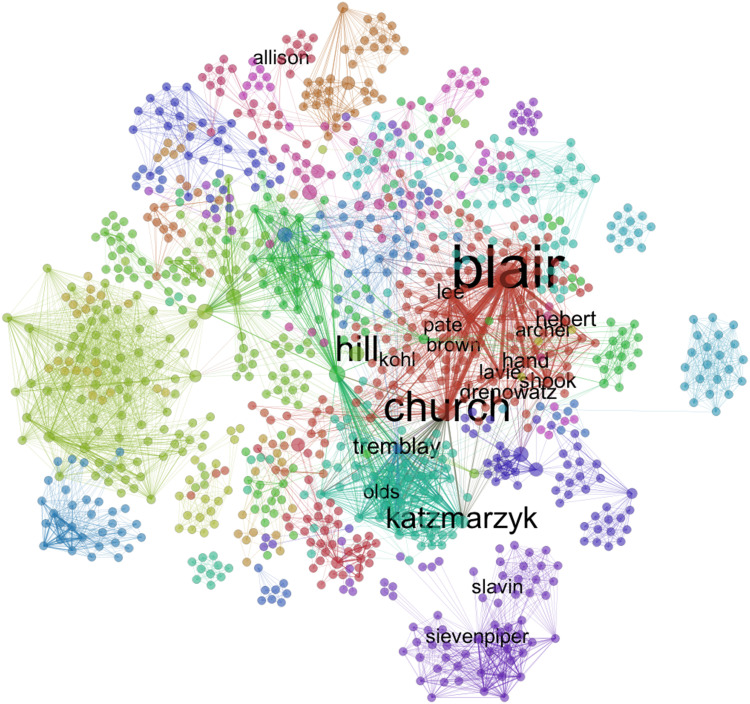
Network of shared Coca-Cola funded publications. Nodes are authors, edges represent co-authored publications and are sized by the number of co-authored publications between two nodes. Nodes are coloured by the edge-betweenness community structure algorithm (explained in text); labels represent a network clique of Coca-Cola funded researchers, identified in personal correspondence between academics and Coca-Cola officials obtained through freedom of information requests

The large number of Coca-Cola funded studies co-authored by the small group of academics in Table 1 suggests they ought to be central nodes in the network of Coca-Cola funded research. To visualize their centrality, we plotted a network of co-authorships using data from Sample 2, restricted to research directly funded by The Coca-Cola Company, The Coca-Cola Foundation, Coca-Cola North America and the Beverage Institute for Health and Wellness, thus excluding any subsidiaries or bottlers outside the USA (see Fig. 4).

In addition to the community search algorithm we ran in this network, as explained in the ‘Analysis’ section, we also applied labels to researchers who were part of a closely affiliated group of academics (with strong ties to Coca-Cola) that was identified in email communications with Coca-Cola officials obtained via requests under states’ open records laws (P Matos Serodio, G Ruskin, M McKee *et al*., unpublished results). This offers an exogenous benchmark to evaluate the performance of the network community structure algorithm and to establish the validity of using co-authorship data on Coca-Cola funded publications to shed light on the company’s involvement in funding scientific research. The size of the labels varies with a measure of node betweenness centrality, which shows the number of times a researcher serves as a bridge between any other two researchers in the network – this gives us an idea of how important they are in controlling the flow of information in the network.

The coloured factions portray different research groups funded by Coca-Cola. Their location in the graph may be driven by geographical factors (such as university affiliation) and by area of research – some authors may focus on physical activity while others work on consumption of non-nutritive sweeteners. Examples of such researchers include Joanne Slavin and John Sievenpiper who focus their research on sweeteners; this moves them close together, but far from the core of the network, which is more attentive to topics of physical activity. At the same time, Sievenpiper’s affiliation to the University of Toronto pushes him away from the core of the network, mostly based in the USA.

The match between coloured subgroups in the core of the network and the location of researchers in close contact with Coca-Cola suggests this group of academics was at the heart of the company’s involvement in funding research, and in a position to participate and coordinate studies between different research groups across fields and across borders, connecting otherwise disconnected groups in the graph.

**Fig. 5:**
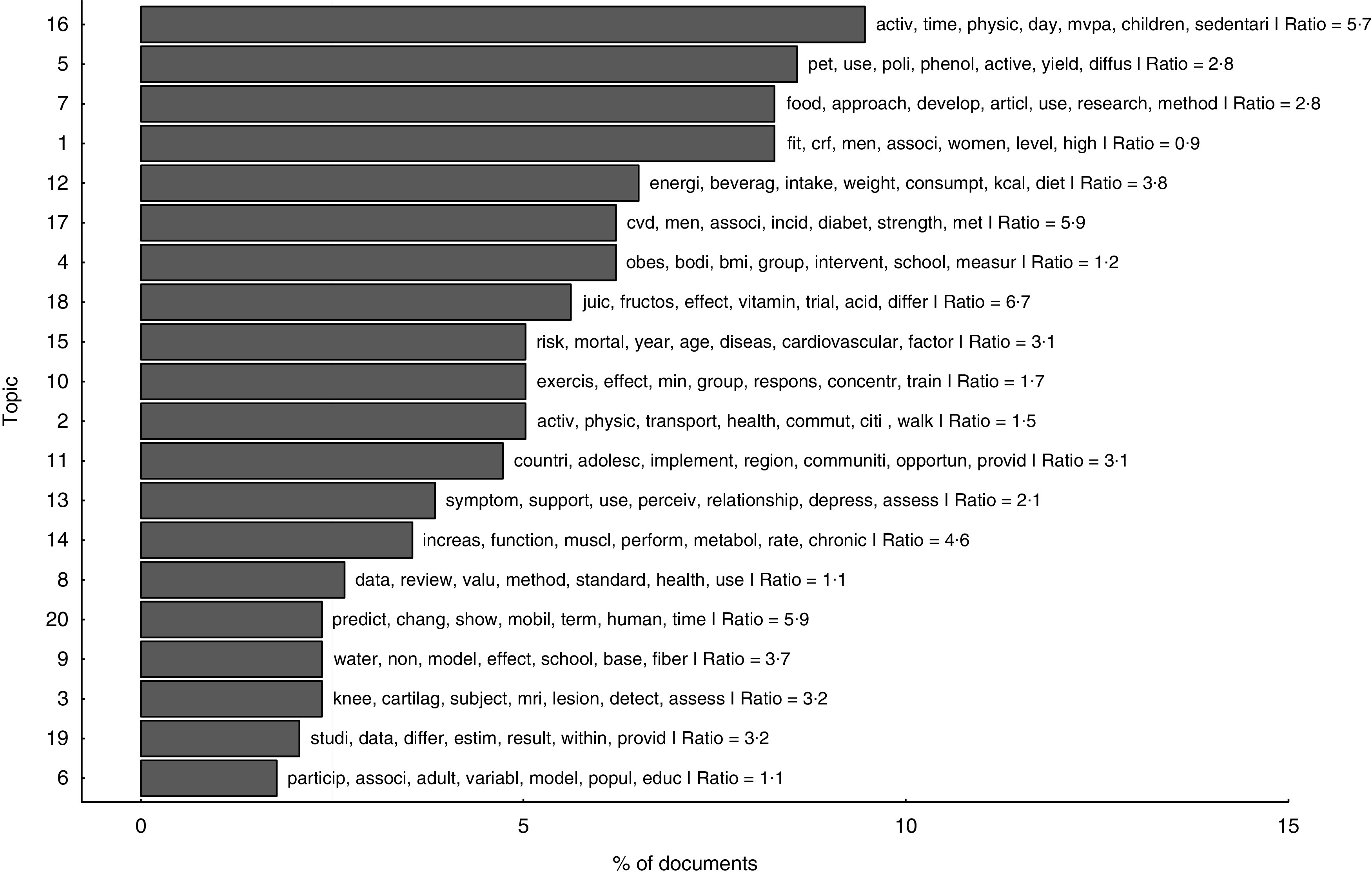
Distribution of topics in the included literature. Based on an analysis of 389 documents from the structural topic model, this graph shows the percentage of documents assigned to each topic. In a way, it measures the topic’s popularity within the corpus of abstracts we retrieved from Web of Science. It is important to note that, although each abstract was assigned to a single topic in this graph (the most probable topic), they are considered a mixture of topics; however, they often devote more words to a particular topic and the algorithm used that information to assign the text to a single topic. The ratio value denotes, for each abstract, how dominant was the most probable topic *v*. the second most probable topic; we averaged these out over all abstracts assigned to each topic in the figure. For example, a ratio of 5·7 for topic 16 means that, on average, the weight of topic 16 in those abstracts assigned to it was 5·7 times larger than the second most probable topic in these abstracts. The twenty word stems with highest probability per topic are listed in Supplemental Table 10 in online supplementary material 3. An interactive visualization of the twenty estimated topics is available in Supplemental Fig. 5 in online supplementary material 3

Turning to the themes of the research, we used structural topic modelling. Figure 5 shows the distribution of topics over documents and the seven most probable words for each topic. As shown, topics converge on physical activity, energy intake, weight, diabetes, exercise and obesity, which are central themes in Coca-Cola’s effort to advance a research agenda able to counteract the link between sugar consumption and obesity by providing a secondary mechanism: the lack of physical activity leading to energy imbalance. Energy balance, physical activity, diabetes and obesity topics account for over 50 % of the studies we analysed (for an interactive visualization of the twenty topics estimated see online supplementary material 3, Supplemental Fig. 5).

Finally, we evaluate the influence of the Coca-Cola funded publications based on journal impact factors. Table 2 shows the top fifteen journals publishing the greatest numbers of Coca-Cola funded studies. This included *Medicine and Science in Sports & Exercise* (twenty-one articles) and other high-ranked journals such as the *American Journal of Clinical Nutrition*, *British Journal of Sports Medicine* and *Journal of the American Medical Association*. The first of these is published by the American College of Sports Medicine, a recipient of substantial funding from Coca-Cola^(^ ^26^ ^)^. These respected journals lend these studies both credibility and visibility within the academic community.

**Table 2:** Selected journals of publication of the 389 Coca-Cola funded articles in Sample 2

Journal	No. of articles	Impact factor
*Medicine and Science in Sports and Exercise*	21	4·141
*American Journal of Clinical Nutrition*	12	6·926
*International Journal of Behavioural Nutrition and Physical Activity*	12	4·396
*PLoS One*	12	2·806
*BMC Public Health*	11	2·265
*Polymer*	11	3·364
*American Journal of Preventive Medicine*	9	4·020
*Mayo Clinic Proceedings*	9	6·686
*Nutrients*	8	3·550
*British Journal of Sports Medicine*	7	6·724
*Obesity (Silver Spring)*	7	3·873
*British Journal of Nutrition*	6	3·706
*Journal of the American College of Cardiology*	6	19·896
*Lancet*	2	44·002
*Journal of the American Medical Association* (*JAMA*)	1	44·405

Sample 2 includes only those studies funded by The Coca-Cola Company and its affiliates in the USA, France, Germany, Spain, New Zealand and Australia, the only countries to release records of their research funding efforts in the form of ‘Transparency Lists’ of funded scientific experts, which were released in late 2015 and early 2016 (see full lists in online supplementary material 1). Sample 2 was compiled by the authors using data retrieved from the Web of Science Core Collection.

### Testing whether Coca-Cola funded researchers declare conflicts of interest

Next we ask whether the academics and scientific experts who are acknowledged by the company as recipients of research funds declare their ties to the company in research publications.

When matching the names of researchers and scientific experts on Coca-Cola’s list to our sample from Web of Science, we failed to account for 176 scientific experts (this includes people who were funded but may not necessarily be academics). These were persons whom Coca-Cola declared as recipients of funding but did not appear in our sample (note we were able to match forty-two names). Possible explanations for this anomaly are that the funded researchers did not disclose their funding sources (whether intentionally or not), disclosed them but the journal did not publish them, did not publish in indexed journals, or were not active academic researchers. To address the last possibility, we then restricted the search further to only those listed on Coca-Cola’s list whom we also were able to confirm were academic researchers (affiliated with an academic institution or actively involved in peer-reviewed research). This yielded thirty-eight confirmed academics. Searching through each of their entire publications’ entries in Web of Science, we were still unable to find any declared conflicts of interest.

## Discussion

Our analysis employed a novel instrument to map the scale, type and persons involved in Coca-Cola’s research networks. It makes a series of important observations. First, it revealed that Coca-Cola’s transparency lists released in the USA^(^ ^6^ ^,^ ^7^ ^)^, the UK^(^ ^8^ ^)^, Australia^(^ ^11^ ^)^, New Zealand^(^ ^12^ ^)^, France^(^ ^9^ ^)^, Germany^(^ ^10^ ^)^ and Spain^(^ ^13^ ^)^ (see online supplementary material 1, Supplemental Tables 5 to 9) are far from complete. There were 471 authors in 128 studies declaring Coca-Cola funding whose names did not appear in any of the transparency lists. A further thirty-eight researchers were on Coca-Cola’s lists, but their publications indexed in Web of Science failed to declare Coca-Cola funding or any conflict of interest. Second, the topical modelling reveals a pattern of consistent themes across the research publications funded by Coca-Cola, emphasizing physical activity over sugar or energy intake in relation to weight gain, diabetes and obesity.

### Limitations of the study

Before interpreting the findings further, we must note several limitations arising from the nature of the data used. First, funding statements rarely identify the principal investigator (or co-investigator) on a grant, which in this case could overestimate the number of authors who appear to have a direct tie to the company. To address this issue, we surveyed the lead authors in each study that acknowledged funding from Coca-Cola and, in the cases where the lead author denied being involved on a Coca-Cola grant, we inquired who were the principal and co-investigators on the grant.

Second, funding statements rarely report the year in which the grant was awarded. It is possible that some studies were awarded grants by the company prior to 2010 but were published only post 2010, which may explain why the author(s) did not appear on Coca-Cola’s transparency lists. However, looking at a subset of articles published in the period 2012–2015, we still found over 400 authors declaring funding from Coca-Cola who were not acknowledged by the company. Furthermore, fourteen authors, involved in twenty studies, confirmed receiving Coca-Cola funding directly to us in the period 2010 to 2015 but were not on Coca-Cola’s lists.

Third, Web of Science only started indexing funding statements for articles published in 2008, which makes this review a small subset of the overall population of Coca-Cola funded studies. However, this asymmetry of information may lead only to an underestimation of the number of researchers funded by Coca-Cola. In addition, our approach is also limited by the fact that Web of Science is currently the only database indexing funding statements in a systematic way, which restricts the literature available for searching (see Appendix 2 for more details).

Fourth, Coca-Cola has amended its transparency lists multiple times. Using website crawling services that store digital archives of the web, we have found that Coca-Cola changed its list of ‘Research and Partnerships’ in the USA at least four times between October 2015 and March 2016. The last change was officially acknowledged by the company as the ‘first update’ to its public disclosure of financial support of scientific research. The update deleted five and added eleven new names to the lists of ‘Health Professionals and Scientific Experts’ and ‘Research and Partnerships’. The results reported above were updated to include the most recent version of all lists published online.

Fifth, authors may incorrectly report funding from The Coca-Cola Company, when it fact it was awarded by a subsidiary or bottler, or indeed awarded by Coca-Cola but to their affiliated institution, not directly funding their publication. Additionally, some researchers may have refused to list their names on Coca-Cola’s website – and although Coca-Cola acknowledges this issue on its website, it also reveals the total amount of funding that was allocated to said researchers was, on aggregate, relatively small: ‘Several individuals with whom we worked in the past have declined to have their names listed. The aggregate amount of funding provided to these individuals over the past five years is approximately $38,000’^(^ ^6^ ^)^.

Finally, it is possible that there are authors we have missed who were not on Coca-Cola’s list and did not disclose funding in their publications; omission of funding source is, unfortunately, difficult to observe and quantify, making our results likely to be conservative estimates of Coca-Cola’s apparent lack of transparency.

## Conclusions

These observations have important implications for managing potential conflicts of interest in research funding. We have learnt from past research that grants from corporations in the tobacco, alcohol, pharmaceutical and gambling industries can have significant effects on the results of published scientific research, although this influence is often denied by those recipients of the industry financial support^(^ ^27^ ^)^. A recent systematic review of systematic reviews on the relationship between sugar-sweetened beverages and weight gain found that industry-sponsored studies were five times more likely to produce results favourable to the companies^(^ ^28^ ^)^. Even in cases where the authors have complete independence to design, implement and analyse the results of a study, the conflict of interests created by industry funding may be enough to compromise the integrity of the conclusions (a recent Cochrane review concluded that standard ‘risk of bias’ assessments could not explain the bias found in pharmaceutical industry-sponsored studies, which suggests a ‘funding bias’ may be a better predictor)^(^ ^29^ ^)^. In the worst-case scenario, bias is introduced to the study design and selection of hypotheses^(^ ^30^ ^,^ ^31^ ^)^.

For policy, our results suggest a general lack of transparency both among funders and researchers. Among industry, despite ostensible efforts of transparency, there remains a significant portion of Coca-Cola funded research that appears to be in the dark. Prior to September 2015, when Coca-Cola published its first transparency list of funded research and partnerships, it is hard to imagine that the public health community and the public at large were fully informed on the extent of Coca-Cola’s involvement in funding research. In this paper, we have demonstrated that even after an important step towards transparency taken by the company, we still know very little about the full scale of Coca-Cola’s funding efforts, let alone of the entire soft drinks industry.

Turning to researchers, our results are consistent with two publicised cases of researchers apparently failing to declare conflicts. One such case involved Jeff Coombes, a professor at the Centre for Research in Exercise, Physical Activity and Health, from the University of Queensland, whose research focuses on using exercise to treat metabolic syndrome. In February of 2016, the Coca-Cola Company reported that he received an ‘unrestricted gift’ of $US 100 000 from the company in 2014 to ‘support ongoing research investigating the effect of exercise intensity on Metabolic Syndrome’^(^ ^32^ ^)^. However, out of sixty-one publications of Professor Coombes between 2014 and 2016 that are indexed in Web of Science,* the Coca-Cola Company is acknowledged as a funding source on only four occasions, the first in May 2016 (article accepted in April 2016)^(^ ^33^ ^)^, three months after Coca-Cola publicly disclosed the details of Coombes’ grant and the press coverage it generated^(^ ^32^ ^)^. In fact, the funding statement in the article provides a link to the webpage of Coca-Cola Australia’s transparency list.

Another involved Fabrice Bonnet, a diabetes researcher at the Institute for European Expertise in Physiology (IEEP), who led a study between 2012 and 2014 to determine whether daily consumption of sweeteners included in carbonated soft drinks affected insulin sensitivity. Bonnet reported funding from the IEEP when registering a clinical trial on 8 January 2014 entitled ‘Comparison of the Effects of a 12-Week Consumption of Two Carbonated Beverages on Insulin Sensitivity’^(^ ^34^ ^)^. However, The Coca-Cola Company acknowledged in December 2016, on its French transparency disclosure^(^ ^9^ ^)^, having granted €719 000 to the IEEP for a ‘research project on intense sweeteners’, for the period 2010 to 2014, which comprises the entire length of Bonnet’s study, according to the registered clinical trial^(^ ^34^ ^)^. Bonnet’s clinical trial did not acknowledge the financial support to the IEEP provided by Coca-Cola.

Such a lack of openness calls for reform and consideration of alternative approaches for managing potential conflicts. Currently debates are being held about the involvement of tobacco industries in e-cigarette research^(^ ^35^ ^)^ and some academic journals have taken the strong measure of banning tobacco industry-funded studies altogether, arguing that they should be viewed as ‘marketing’ for the industry^(^ ^36^ ^)^. Our findings suggesting a lack of transparency in an industry that has claimed to be fully open, contribute to a climate of distrust. This may warrant the beginnings of a conversation about similar restrictions on research funded by the sugar and related industries.

Box Research highlights

• There is concern in public health that The Coca-Cola Company may fund research that benefits its corporate interests and diverts attention from the role of sugar-sweetened beverages in the obesity epidemic. • In 2015, The Coca-Cola Company published several lists of health professionals, scientific experts and academic researchers with whom it collaborated and whose research it funded between 2010 and 2015. It is not clear whether these lists are comprehensive. • The Coca-Cola Company, in conjunction with The Coca-Cola Foundation and the Beverage Institute for Health and Wellness, has funded 389 studies between 2008 and 2016, published in 169 journals, involving more than 1000 authors. • Although Coca-Cola took a step towards transparency, our data have shown major gaps and errors in its disclosures of research funding: Coca-Cola has acknowledged only forty-two out of 513 potential investigators on grants awarded by the company. • Coca-Cola predominantly funds research on nutrition, with a focus on physical activity, the concept of ‘energy balance’ and how these two factors relate to obesity and diabetes.

## Footnotes

* Note that the Canadian Diabetes Association became Diabetes Canada on 13 February 2017^(^ ^25^ ^)^.

## Supplementary material

For supplementary material accompanying this paper visit https://doi.org/10.1017/S136898001700307X. click here to view supplementary material

## Appendix 1

***Literature searches***

1. Web of Science search for systematic review using Core Collection database: ft=“cola”
2. Web of Science search for variants of ‘Coca-Cola’: fo=“coca-cola” OR fo=“coca cola” OR fo=“coco-cola” OR fo=“cocacola” OR fo= “beverage health & wellness institute” OR fo=“beverage institute for health & wellness” OR fo=“beverage institute for health wellness” OR fo=“beverage institute for health and wellness”

## Appendix 2

***Limitations of Web of Science funding acknowledgement data***

The use of Thomson Reuters’ Web of Science Core Collection to study funding sources in academic research is not new^(^ ^37^ ^,^ ^38^ ^)^. However, important limitations have been raised in recent work^(^ ^39^ ^,^ ^40^ ^)^.

Thomson Reuters began to index information contained in the funding acknowledgement section of articles in 2008. This means that the first year with substantial coverage of funding information in published research is 2009. The database also presents other smaller caveats: (i) funding acknowledgement data are covered mainly for the Science Citation Index Expanded database, and not for Social Science Citation Index or Arts and Humanities Citation Index databases; (ii) information is indexed only for publications where the funding text is in English; and (iii) it only indexes information where the paratext includes mentions of ‘funding’ (other kinds of support are ignored).

For our purposes, these are minor caveats, except for the fact that the sample is truncated, since we can look at only a small window of Coca-Cola funding activities because data before 2008 are missing.

However, other concerns have also been raised about the way in which the algorithm determines whether the company listed in the funding text is indeed a funding agency for that study. Lewison and Sullivan^(^ ^40^ ^)^ find that the algorithm over-classifies companies as the funding agencies in situations where they are simply included for other monetary arrangements with an author which would usually fall under a conflict of interest, and not a funding contract for that particular study.

We worked around this by reading the funding paratext for each publication and excluding those articles where Coca-Cola is (mistakenly) listed as a funding agency because there is simply a conflict of interest reported in the funding acknowledgement.

Furthermore, another caveat we should note is the inconsistency of use of the author strings across publications. The use of middle names, or first name initials, changes frequently across publications. To illustrate, S. Blair, Steven Blair, S N Blair, Steven N Blair are variants of the same author’s name used across publications. We standardize the author name strings for all documents to avoid duplicates in our analysis.
