# Supplementary material for: Coca-Cola – a model of transparency in research partnerships? A network analysis of Coca-Cola’s research funding (2008–2016)
Source: Public Health Nutr. 2018 Mar 21;21(9):1594–607. doi: 10.1017/S136898001700307X (PMC5962884; doi:10.1017/S136898001700307X)
Supplement: Supplementary file 1 [file S136898001700307Xsup001.docx]

**Web Appendix**

**Table 1:** Top 15 affiliations listed in our sample of Coca-Cola funded articles.

| Rank | University | # Articles |
| --- | --- | --- |
| 1 | University of South Carolina | 89 |
| 2 | Louisiana State University | 75 |
| 3 | Pennington Biomedical Research Center | 74 |
| 4 | University of North Carolina | 27 |
| 5 | University of Queensland | 27 |
| 6 | University of Georgia | 25 |
| 7 | Iowa State University | 22 |
| 8 | University of Ottawa | 21 |
| 9 | Georgia Institute of Technology | 20 |
| 10 | University of California | 20 |
| 11 | Arizona State University | 18 |
| 12 | Emory University | 16 |
| 13 | University of the Andes Colombia | 16 |
| 14 | University of Alabama | 15 |
| 15 | University of Bath | 15 |

**Source:** Compiled by the authors using data from the Web of Science Core Collection.

**Table 2:** List of health professionals and scientific experts based in the USA that collaborated with The Coca-Cola Company between 2010 and 2015.

| Name |
| --- |
| Adam Drewnowski |
| Adam Seidner |
| Alisa Winters |
| Alison Eastwood |
| Allison Topilow |
| Alyson Mace |
| Alyssa Rider Corell |
| Amia Freeman |
| Amy Bragg |
| Angela Medearis |
| Angela Stewart Goka |
| Angelica Millan |
| Angie Cason |
| Audwin Fletcher |
| Bennett Weinberg |
| Bernadene Magnuson |
| Betsy Hornick |
| Carl Lavie |
| Carol Berg Sloan |
| Carol Meerschaert |
| Chrissy Barth |
| Christina Meyer-Jax |
| Cristina Rivera |
| Daniel Santibanez |
| Danielle McCauley |
| David Vanata |
| Deanna Latson |
| Debbie Mouser |
| Deborah Enos |
| Diane Greenleaf-Kisner |
| Donna Manring |
| Donna Shields |
| Elaine Magee |
| Elba González Pérez |
| Elizabeth Applegate |
| Elizabeth Patton |
| Eric Cochran |
| Gail Posner |
| Gayle Jennings |
| Gregory Hand |
| Heidi McIndoo |
| Jacqueline Gomes |
| Jae Berman |
| James Hill |
| Jamie Seidner |
| Jan Tilley |
| Jay Cardiello |
| Jerrod Libonati |
| Jessica Levinson |
| John Calfee |
| John Foreyt |
| John Sievenpiper |
| Judith Feola Gordon |
| Julie Feldman |
| Kathryn Fink |
| Kathy Warwick |
| Katie Cavuto Boyle |
| Kim Galeaz |
| Kimberly Evans |
| Kimberly Grabarz |
| Kristen Marshall |
| Kristen Smith |
| Laura Ortiz |
| LeeAnn Weintraub |
| Lindsey Joe |
| Lisa Jones |
| Lisa Moskovitz |
| Lora Burke |
| Luke Corey |
| Marisa Persky |
| Marissa Kelley |
| Mark Haub |
| Martha McHenry |
| Martha Rosenau |
| Mary Beth Knight |
| Mary Zupke |
| Melissa Hermann Dierks |
| Michele Reed |
| Michelle May |
| Michelle Rowe |
| Michelle Stewart |
| Molly Gee |
| Mona Rosene |
| Naomi Kakiuchi |
| Natalie Webb |
| Nicole Fasules |
| Norma Rixter |
| Pamela Peeke |
| Pat Baird |
| Peter Martin |
| Philip Goglia |
| Rachel Brandeis |
| Ramona Braganza |
| Rani Whitfield |
| Renee Clerkin |
| Renee Simms |
| Rima Kleiner |
| Rita Ng |
| Robert Murray |
| Robert Sallis |
| Roberta Schwartz Wennik |
| Robyn Flipse |
| Robyn Kievit |
| Roger Clemens |
| Ronnie Woo |
| Sarah Jane Bedwell |
| Sophia Kamveris |
| Stacey Matthews-Woodson |
| Steven Blair |
| Sylvia Meléndez Klinger |
| Theresa Gentile |
| Victoria Shanta Retelny |
| Virginia Willis |
| Wendy Kohrt |
| Yvette Rooks |

Source: The Coca-Cola Company website.

**Table 3**: List of researchers that received health and wellbeing research grants from Coca-Cola between 2010 and 2015, for projects based in the USA. Some of the amounts listed refer to joint grant collaborations that involved more than one researcher on the list. This information was retrieved from The Coca-Cola Company’s website.

| Name | Institution | Year | Amount ($US) |
| --- | --- | --- | --- |
| Anura Kurpad | St. Johns Research Institute | 2010-2014 | 6,426,308 |
| Carol Maher | University of South Australia | 2010-2014 | 6,426,308 |
| Caterine Tudor-Locke | Pennington Biomedical Research Center | 2010-2014 | 6,426,308 |
| Estelle V Lambert | University of Cape Town | 2010-2014 | 6,426,308 |
| Jean-Philippe Chaput | Children’s Hospital of Eastern Ontario Research Institute | 2010-2014 | 6,426,308 |
| José Maia | University of Porto | 2010-2014 | 6,426,308 |
| Mark Tremblay | Children’s Hospital of Eastern Ontario Research Institute | 2010-2014 | 6,426,308 |
| Martyn Standage | University of Bath | 2010-2014 | 6,426,308 |
| Mikael Fogelholm | University of Helsinki | 2010-2014 | 6,426,308 |
| Olga L Sarmiento | Universidad de los Andes | 2010-2014 | 6,426,308 |
| Pei Zhao | Tianjin Women’s and Children’s Health Center | 2010-2014 | 6,426,308 |
| Peter T Katzmarzyk | Pennington Biomedical Research Center | 2010-2014 | 6,426,308 |
| Rebecca Kuriyan | St. Johns Research Institute | 2010-2014 | 6,426,308 |
| Tim Church | Pennington Biomedical Research Center | 2010-2014 | 6,426,308 |
| Timothy Olds | University of South Australia | 2010-2014 | 6,426,308 |
| Victor Matsudo | Centro de Estudos do Laboratório de Aptidão Física de São Caetano do Sul | 2010-2014 | 6,426,308 |
| Vincent Onywera | Kenyatta University | 2010-2014 | 6,426,308 |
| Steven Blair | University of South Carolina | 2010 | 4,409,000 |
| Venkat Narayan | Emory University | 2010-2012 | 1,851,000 |
| James Rippe | Rippe Lifestyle Institute | 2013-2015 | 1,220,786 |
| Gregory Hand | University of South Carolina | 2010-2013 | 851,000 |
| Steven Blair | University of South Carolina | 2010-2013 | 851,000 |
| David Allison | University of Alabama | 2010-2014 | 335,000 |
| Marc Hamilton | Pennington Biomedical Research Foundation | 2011-2014 | 319,000 |
| Michelle Althuis | Lincoln Greystone | 2010-2014 | 319,000 |
| David Allison | University of Alabama | 2011 | 299,000 |
| Neil Johannsen | Pennington Biomedical Research Center | 2010-2011 | 266,602 |
| Tim Church | Pennington Biomedical Research Center | 2010-2011 | 266,602 |
| Charlene Bayer | Georgia Institute of Technology | 2010-2011 | 259,000 |
| Mindy Millard-Stafford | Georgia Institute of Technology | 2010-2011 | 259,000 |
| Douglas Weed | DLW Consulting Services | 2010-2015 | 168,000 |
| Steven Blair | University of South Carolina | 2010-2014 | 167,000 |
| John Foreyt | Baylor College of Medicine | 2013 | 161,000 |
| Matthew S. Ganio | University of Arkansas | 2012-2014 | 145,000 |
| Venkat Narayan | Emory University | 2014 | 145,000 |
| Mindy Millard-Stafford | Georgia Institute of Technology | 2010 | 121,000 |
| John Sievenpiper | University of Toronto | 2014 | 112,000 |
| Mindy Millard-Stafford | Georgia Institute of Technology | 2010-2011 | 105,000 |
| Tim Church | Pennington Biomedical Research Foundation | 2010 | 100,000 |
| John Sievenpiper | Toronto 3D Knowledge Synthesis and ClinicalTrials Foundation | 2014 | 80,000 |
| Tim Church | Pennington Biomedical Research Foundation | 2010 | 80,000 |
| Susan Jebb | Medical Research Council | 2010 | 77,000 |
| Corby Martin | Pennington Biomedical Research Center | 2012-2015 | 76,747 |
| James Rippe | Rippe Lifestyle Institute | 2011 | 76,574 |
| Harvey Anderson | University of Toronto | 2014 | 50,000 |
| Steven Blair | University of South Carolina | 2011 | 50,000 |
| Lynn Bailey | University of Georgia | 2014 | 45,000 |
| Richard Lewis | University of Georgia | 2014 | 45,000 |
| Mary Ann Johnson | University of Georgia | 2014-2015 | 37,000 |
| Venkat Narayan | Emory Global Diabetes Research Center | 2015 | 35,000 |
| Brent Hutto | University of South Carolina | 2013 | 32,000 |
| Joanne Slavin | University of Minnesota | 2010-2015 | 25,000 |
| Adam Drewnowski | University of Washington Foundation | 2010-2015 | 250,00 |

Source: The Coca-Cola Company website.

**Table 4**: Health and Wellbeing Research and Partnerships in the UK (list of researchers)

| Name |
| --- |
| Dr. Margaret Ashwell |
| Sue Baic |
| Prof. Stuart Biddle |
| Prof. Nino Binns |
| Sean Blair |
| Prof. Alan Boobis |
| Debbie Cook |
| Prof. Monty Duggal |
| Fiona Hunter |
| Prof. Ken Fox |
| Lynne Garton |
| Dr. Geoffrey Livesey |
| Dr. Sigrid Gibson |
| Dr. David Haslam |
| Prof. Marion Hetherington |
| Penny Hunking |
| Angie Jefferson |
| Prof. Ian Macdonald |
| Dr. Nick Marcks |
| Prof. Ronald Maughan |
| Sian Porter |
| Prof. Andy Renwick |
| Prof. Peter Rogers |
| Dr. Carrie Ruxton |
| Carol Weir |
| Prof. Greg Whyte |
| Dr. Zoe Williams |

Source: The Coca-Cola Great Britain website.

**Table 5**: List of Health Professionals & Scientific Experts in Australia.

| Name | Descriptor |
| --- | --- |
| Dr Alan Barclay, PhD | Webinar presentation for Health Professionals 2011 |
| Louise Burke | Webinar presentation for Health Professionals 2010 |
| Glenn Cardwell AdvAPD | Member of Coca-Cola South Pacific Health & Wellness Advisory Council 2013-2014 and Paper Review 2015* |
| Professor Peter Clifton | Member of Coca-Cola South Pacific Health & Wellness Advisory Council 2010-2014 and Paper Review 2015* |
| Bernadette Drummond | Member of Coca-Cola South Pacific Health & Wellness Advisory Council and Paper Review 2015* |
| Dr G K Harinath | Member of Coca-Cola South Pacific Health & Wellness Advisory Council 2013* |
| Dr Gina Levy | Presentation of research at Symposium 2013 |
| Erin Mahoney | Member of Coca-Cola South Pacific Health & Wellness Advisory Council 2010-2014* |
| Sharon Natoli APD | Member of Coca-Cola South Pacific Health & Wellness Advisory Council 2010-2011* |
| Professor Paul Nestel | Member of Coca-Cola South Pacific Health & Wellness Advisory Council 2010-2014 and Paper Review 2015* |
| Dr Nancy J Rehrer | Member of Coca-Cola South Pacific Health & Wellness Advisory Council 2010-2014* |
| Bill Shrapnel | Facilitator at Symposium 2013 |
| Zoe Wilson APD | Nutrition contributor website 2015 |
| Dr Alan Barclay, PhD | Webinar presentation for Health Professionals 2011 |
| Louise Burke | Webinar presentation for Health Professionals 2010 |
| Glenn Cardwell AdvAPD | Member of Coca-Cola South Pacific Health & Wellness Advisory Council 2013-2014 and Paper Review 2015* |
| Professor Peter Clifton | Member of Coca-Cola South Pacific Health & Wellness Advisory Council 2010-2014 and Paper Review 2015* |
| Bernadette Drummond | Member of Coca-Cola South Pacific Health & Wellness Advisory Council and Paper Review 2015* |
| Dr G K Harinath | Member of Coca-Cola South Pacific Health & Wellness Advisory Council 2013* |
| Dr Gina Levy | Presentation of research at Symposium 2013 |
| Erin Mahoney | Member of Coca-Cola South Pacific Health & Wellness Advisory Council 2010-2014* |
| Sharon Natoli APD | Member of Coca-Cola South Pacific Health & Wellness Advisory Council 2010-2011* |
| Professor Paul Nestel | Member of Coca-Cola South Pacific Health & Wellness Advisory Council 2010-2014 and Paper Review 2015* |
| Dr Nancy J Rehrer | Member of Coca-Cola South Pacific Health & Wellness Advisory Council 2010-2014* |
| Bill Shrapnel | Facilitator at Symposium 2013 |
| Zoe Wilson APD | Nutrition contributor website 2015 |
| Dr Alan Barclay, PhD | Webinar presentation for Health Professionals 2011 |

Source: The Coca-Cola Australia website.

**Table 6**: List of Health Professionals & Scientific Experts in Germany.

| Name |
| --- |
| Lena Becker |
| Prof. Dr. Wolf-Dietrich Brettschneider |
| Mia Buhler |
| Andreas Dittbemer |
| Sarah Eichhorn |
| Prof. Dr. Klaus Fischer |
| Anna Frost |
| Verena Frost |
| Karin Grüttner |
| Kirsten Metternich |
| Vanessa sleeve |
| Evelyn Knecht |
| Stefan Koffinke |
| Prof. Dr. Dr. hc Berthold Koletzko |
| Anja Krumbe |
| Lina Mallon |
| Nadine Mellert |
| Linda Menke |
| Margret Morlo |
| Sven-David Mueller |
| Daniel Pammé |
| Prof. Dr. Dr. Regitz-Zagrosek |
| Prof. Dr. Una M. Röhr-Sendlmeier |
| Britta Steffen |
| Ann-Christin Weber |
| Laura Werner |
| Dr. Eva Wlodarek |
| Prof. Dr. Hans Peter Brandl-Bredenbeck |
| Dr. Ursula Wölwer-Rieck |

Source: The Coca-Cola Germany website.

**Table 7**: List of Health Professionals & Scientific Experts in France.

| Name | Descriptor |
| --- | --- |
| France Bellisle | Docteur en Sciences |
| Xavier Bigard | Professeur physiologiste nutritionniste |
| François Carré | Professeur cardiologue et médecin du sport |
| Arnaud Cocaul | Médecin nutritionniste |
| Richard C. Delerins | Ph.D. anthropologue EHESS Paris |
| Charles-Yannick Guezennec | Médecin du sport |
| Bernard Guy-Grand | Professeur des universités honoraire |
| Pascale Hébel | Directrice du Pôle Consommation et Entreprise au CREDOC |
| Emmanuelle Lecuyer | Diététicienne nutritionniste |
| Sandrine Leroux | Diététicienne nutritionniste |
| Thérèse Libert | Diéteticienne nutritionniste |
| Pascale Modaï | Médecin nutritionniste |
| Christine Rodier | Anthropologue Université de Lausanne |
| Claire Rousseau | Diététicienne nutritionniste |
| Jean-Louis Schlienger | Professeur honoraire de la faculté de médecine de Strasbourg |
| Gabriel Tavoularis | Expert de la consommation alimentaire au CREDOC |
| Julie Viel | Diététicienne nutritionniste |
| Bernard Wayslfeld | Médecin nutritionniste et psychiatre |

Source: The Coca-Cola France website.

**Table 8**: Research consortia/Organizations with funding links to Coca-Cola, as recovered from Web of Science, that were excluded from the Web of Science sample during the screening steps (see Figure 1 in the article).

| Consortia/Organization |
| --- |
| International Study on Childhood Obesity, Lifestyle and Environment (ISCOLE) |
| Global Alliance for Improved Nutrition (GAIN) |
| Global Energy Balance Network (GEBN) |
| International Life Sciences Institute (ILSI) |
| ARAB-EAT project |
| Epode Umbria Region Obesity Prevention Study (EUROBIS) |
| Latin American Study of Nutrition and Health (ELANS) |
| Centre de recherche pour l'étude et l'observation des conditions de vie (CRÉDOC) |
| International Prevention Research Insitute (iPRI) |
| Pennington Biomedical Research Center |
| Kidney Disease: Improving Global Outcomes (KDIGO) |
| Research Group of Physical Activity and Quality of Life (GPAQ) |
| participACTION Teen Challenge program evaluation study |
| Forum on Child Obesity Interventions (org. by FUNSALUD) |
| American College of Sports Medicine Foundation (ACSM) |
| Academy of Nutrition and Dietetics Foundation |
| Energy Balance Work Group |
| Exercise is Medicine (org. ACSM) |
| Anthropometry, Intake and Energy Balance (ANIBES) |
| Transportation Air Pollution and Physical Activities (TAPAS) |

Source: Compiled by the authors using information from Web of Science;

**Table 9:** Title and year of publication of scientific literature funded by Coca-Cola and included in Sample 1 only, or Sample 1 and 2.

| Title | Year | Sample |
| --- | --- | --- |
| Value-added conversion of waste cooking oil and post-consumer PET bottles into biodiesel and polyurethane foams | 2016 | Sample 1 only |
| Perceived and objective neighborhood support for outside of school physical activity in South African children | 2016 | Sample 1 only |
| Steviol glycosides in purified stevia leaf extract sharing the same metabolic fate | 2016 | Sample 1 only |
| The role of social support in weight loss maintenance: results from the MedWeight study | 2016 | Sample 1 only |
| Accuracy of Heart Rate Watches: Implications for Weight Management | 2016 | Sample 1 only |
| No Change in 24-Hour Hydration Status Following a Moderate Increase in Fluid Consumption | 2016 | Sample 1 only |
| Spatial characterization of the seawater upconing process in a coastal Mediterranean aquifer (Plana de Castellon, Spain): evolution and controls | 2016 | Sample 1 only |
| Increased Rotavirus Prevalence in Diarrheal Outbreak Precipitated by Localized Flooding, Solomon Islands, 2014 | 2016 | Sample 1 only |
| Polyol production during heterofermentative growth of the plant isolate Lactobacillus florum 2F | 2016 | Sample 1 only |
| Education status determines 10-year (2002-2012) survival from cardiovascular disease in Athens metropolitan area: the ATTICA study, Greece | 2016 | Sample 1 only |
| The Influence of Neighborhood Crime on Increases in Physical Activity during a Pilot Physical Activity Intervention in Children | 2016 | Sample 1 only |
| Fructose Containing Sugars at Normal Levels of Consumption Do Not Effect Adversely Components of the Metabolic Syndrome and Risk Factors for Cardiovascular Disease | 2016 | Sample 1 only |
| Beverage Consumption Habits and Association with Total Water and Energy Intakes in the Spanish Population: Findings of the ANIBES Study | 2016 | Sample 1 only |
| Prospective association between body composition, physical activity and energy intake in young adults | 2016 | Sample 1 only |
| Accelerometer measured sedentary behavior and physical activity in white and black adults: The REGARDS study | 2016 | Sample 1 only |
| Effects of clinically significant weight loss with exercise training on insulin resistance and cardiometabolic adaptations | 2016 | Sample 1 only |
| Physical Activity Levels in US Latino/Hispanic Adults Results From the Hispanic Community Health Study/Study of Latinos | 2016 | Sample 1 only |
| Associations Between Parental Perceptions of the Neighborhood Environment and Childhood Physical Activity: Results from ISCOLE-Kenya | 2016 | Sample 1 only |
| Macronutrient Distribution and Dietary Sources in the Spanish Population: Findings from the ANIBES Study | 2016 | Sample 1 only |
| Metabolic syndrome and 10-year cardiovascular disease incidence: The ATTICA study | 2016 | Sample 1 only |
| Anti-inflammatory Dietary Inflammatory Index scores are associated with healthier scores on other dietary indices | 2016 | Sample 1 only |
| Health Impacts of Active Transportation in Europe | 2016 | Sample 1 only |
| Perceived Neighborhood Environmental Attributes Associated with Walking and Cycling for Transport among Adult Residents of 17 Cities in 12 Countries: The IPEN Study | 2016 | Sample 1 only |
| Impact of traffic-related air pollution on acute changes in cardiac autonomic modulation during rest and physical activity: a cross-over study | 2016 | Sample 1 only |
| Metabolic syndrome, adherence to the Mediterranean diet and 10-year cardiovascular disease incidence: The ATTICA study | 2016 | Sample 1 only |
| Effects of a beverage rich in (poly)phenols on established and novel risk markers for vascular disease in medically uncomplicated overweight or obese subjects: A four week randomized placebo-controlled trial | 2016 | Sample 1 only |
| Patterns of Sedentary Behavior in US Middle-Age and Older Adults: The REGARDS Study | 2016 | Sample 1 only |
| Where are all the self-employed women? Push and pull factors influencing female labor market decisions | 2016 | Sample 1 only |
| Physical Activity Patterns of the Spanish Population Are Mostly Determined by Sex and Age: Findings in the ANIBES Study | 2016 | Sample 1 only |
| At the Mercy of the Gods: Associations Between Weather, Physical Activity, and Sedentary Time in Children | 2016 | Sample 1 only |
| Relation of Body's Lean Mass, Fat Mass, and Body Mass Index With Submaximal Systolic Blood Pressure in Young Adult Men | 2016 | Sample 1 only |
| Individual and School-Level Socioeconomic Gradients in Physical Activity in Australian Schoolchildren | 2016 | Sample 1 only |
| Biological conversion of biogas to methanol using methanotrophs isolated from solid-state anaerobic digestate | 2016 | Sample 1 only |
| Integration of decentralized torrefaction with centralized catalytic pyrolysis to produce green aromatics from coffee grounds | 2016 | Sample 1 only |
| Latin American Study of Nutrition and Health (ELANS): rationale and study design | 2016 | Sample 1 only |
| A systematic review and meta-analysis of nut consumption and incident risk of CVD and all-cause mortality | 2016 | Sample 1 only |
| Clustering of Dietary Patterns, Lifestyles, and Overweight among Spanish Children and Adolescents in the ANIBES Study | 2016 | Sample 1 only |
| Subjective Estimation of Physical Activity Using the International Physical Activity Questionnaire Varies by Fitness Level | 2016 | Sample 1 only |
| EATING PATTERNS, PHYSICAL ACTIVITY AND THEIR ASSOCIATION WITH DEMOGRAPHIC FACTORS IN THE POPULATION INCLUDED IN THE OBESITY STUDY IN ROMANIA (ORO STUDY) | 2016 | Sample 1 only |
| Use of Placebo in Pediatric Inflammatory Bowel Diseases: A Position Paper From ESPGHAN, ECCO, PIBDnet, and the Canadian Children IBD Network | 2016 | Sample 1 only |
| Dietary supplementation with purified citrus limonin glucoside does not alter ex vivo functions of circulating T lymphocytes or monocytes in overweight/obese human adults | 2016 | Sample 1 only |
| Adherence to Mediterranean diet and 10-year incidence (2002-2012) of diabetes: correlations with inflammatory and oxidative stress biomarkers in the ATTICA cohort study | 2016 | Sample 1 only |
| An Overweight Preventive Score associates with obesity and glycemic traits | 2016 | Sample 1 only |
| Urban magnetism through the lens of geo-tagged photography | 2015 | Sample 1 only |
| Association Between Objectively Measured Physical Activity and Cognitive Function in Older Adults-The Reasons for Geographic and Racial Differences in Stroke Study | 2015 | Sample 1 only |
| Developing a Culturally Sensitive Lifestyle Behavior Change Program for Older Latinas | 2015 | Sample 1 only |
| Low levels of physical activity are associated with dysregulation of energy intake and fat mass gain over 1 year | 2015 | Sample 1 only |
| The Added Benefit of Bicycle Commuting on the Regular Amount of Physical Activity Performed | 2015 | Sample 1 only |
| The Prospective Association between Different Types of Exercise and Body Composition | 2015 | Sample 1 only |
| Association between cardiorespiratory fitness and submaximal systolic blood pressure among young adultmen: a reversed J-curve pattern relationship | 2015 | Sample 1 only |
| Association between electronic equipment in the bedroom and sedentary lifestyle, physical activity, and body mass index of children | 2015 | Sample 1 only |
| Moderating effects of age, gender and education on the associations of perceived neighborhood environment attributes with accelerometer-based physical activity: The IPEN adult study | 2015 | Sample 1 only |
| Differences in correlates of energy balance in normal weight, overweight and obese adults | 2015 | Sample 1 only |
| Evaluation of a Voluntary Worksite Weight Loss Program on Metabolic Syndrome | 2015 | Sample 1 only |
| Assessment of diet quality improves the classification ability of cardiovascular risk score in predicting future events: The 10-year follow-up of the ATTICA study (2002-2012) | 2015 | Sample 1 only |
| Weight regaining: From statistics and behaviors to physiology and metabolism | 2015 | Sample 1 only |
| High Intensity Interval- vs Moderate Intensity-Training for Improving Cardiometabolic Health in Overweight or Obese Males: A Randomized Controlled Trial | 2015 | Sample 1 only |
| Physical activity training in US medical schools: Preparing future physicians to engage in primary prevention | 2015 | Sample 1 only |
| Association Between Television Viewing and Physical Activity in 10-Year-Old Brazilian Children | 2015 | Sample 1 only |
| Objective correlates and determinants of bicycle commuting propensity in an urban environment | 2015 | Sample 1 only |
| No Effect of Added Sugar Consumed at Median American Intake Level on Glucose Tolerance or Insulin Resistance | 2015 | Sample 1 only |
| Implementing music therapy on an adolescent inpatient unit: a mixed-methods evaluation of acceptability, experience of participation and perceived impact | 2015 | Sample 1 only |
| Physical Activity, Sedentary Time, and Obesity in an International Sample of Children | 2015 | Sample 1 only |
| Utilization and Harmonization of Adult Accelerometry Data: Review and Expert Consensus | 2015 | Sample 1 only |
| Association between actigraphic sleep metrics and body composition | 2015 | Sample 1 only |
| Psychometric properties of the Chinese (Cantonese) versions of the KIDSCREEN health-related quality of life questionnaire | 2015 | Sample 1 only |
| Hierarchical modelling of blood lipids' profile and 10-year (2002-2012) all cause mortality and incidence of cardiovascular disease: the ATTICA study | 2015 | Sample 1 only |
| Standardization of the Food Composition Database Used in the Latin American Nutrition and Health Study (ELANS) | 2015 | Sample 1 only |
| Biological variation of plasma osmolality obtained with capillary versus venous blood | 2015 | Sample 1 only |
| A transcriptional signature of "exercise resistance" in skeletal muscle of individuals with type 2 diabetes mellitus | 2015 | Sample 1 only |
| Alternative food networks and food provisioning as a gendered act | 2015 | Sample 1 only |
| Sexualized Avatars Lead to Women's Self-Objectification and Acceptance of Rape Myths | 2015 | Sample 1 only |
| Pyrolysis of spent coffee grounds using a screw-conveyor reactor | 2015 | Samples 1 & 2 |
| A survey of catalysts for aromatics from fast pyrolysis of biomass | 2015 | Samples 1 & 2 |
| Moderate-to-Vigorous Physical Activity and Sedentary Behavior: Independent Associations With Body Composition Variables in Brazilian Children | 2015 | Samples 1 & 2 |
| Variability and Stability in Daily Moderate-to-Vigorous Physical Activity among 10 Year Old Children | 2015 | Samples 1 & 2 |
| Plasma cytokine and exertional responses in relation to exercise intensity and volume of exercising muscle mass during arm-crank ergometry | 2015 | Samples 1 & 2 |
| Normal or High Polyphenol Concentration in Orange Juice Affects Antioxidant Activity, Blood Pressure, and Body Weight in Obese or Overweight Adults | 2015 | Samples 1 & 2 |
| Weight loss maintenance in relation to locus of control: The MedWeight study | 2015 | Samples 1 & 2 |
| Relationship between lifestyle behaviors and obesity in children ages 9-11: Results from a 12-country study | 2015 | Samples 1 & 2 |
| Brief report: symmetricity of radiographic and MRI-detected structural joint damage in persons with knee pain - the Joints on Glucosamine (JOG) Study | 2015 | Samples 1 & 2 |
| Why Are Children Different in Their Daily Sedentariness? An Approach Based on the Mixed-Effects Location Scale Model | 2015 | Samples 1 & 2 |
| Effect of ethnicity on glycaemic index: a systematic review and meta-analysis | 2015 | Samples 1 & 2 |
| Hydration Status over 24-H Is Not Affected by Ingested Beverage Composition | 2015 | Samples 1 & 2 |
| Exercise and the Cardiovascular System Clinical Science and Cardiovascular Outcomes | 2015 | Samples 1 & 2 |
| Validation of the HellenicSCORE (a Calibration of the ESC SCORE Project) Regarding 10-Year Risk of Fatal Cardiovascular Disease in Greece | 2015 | Samples 1 & 2 |
| Are BMI and Sedentariness Correlated? A Multilevel Study in Children | 2015 | Samples 1 & 2 |
| High prevalence of elevated haemoglobin A1C among adolescent blood donors: Results from a voluntary screening programme including 31,546 adolescents | 2015 | Samples 1 & 2 |
| Autonomous Motivation Predicts 7-Day Physical Activity in Hong Kong Students | 2015 | Samples 1 & 2 |
| Dietary fiber effects in chronic kidney disease: a systematic review and meta-analysis of controlled feeding trials | 2015 | Samples 1 & 2 |
| Influence of the Source of Social Support and Size of Social Network on All-Cause Mortality | 2015 | Samples 1 & 2 |
| Dietary Intake, FTO Genetic Variants, and Adiposity: A Combined Analysis of Over 16,000 Children and Adolescents | 2015 | Samples 1 & 2 |
| Obtaining Accelerometer Data in a National Cohort of Black and White Adults | 2015 | Samples 1 & 2 |
| Chronic administration of a microencapsulated probiotic enhances the bioavailability of orange juice flavanones in humans | 2015 | Samples 1 & 2 |
| Taking High Conservation Value from Forests to Freshwaters | 2015 | Samples 1 & 2 |
| Breaking up of prolonged sitting over three days sustains, but does not enhance, lowering of postprandial plasma glucose and insulin in overweight and obese adults | 2015 | Samples 1 & 2 |
| Accelerometer-based physical activity levels among Mexican adults and their relation with sociodemographic characteristics and BMI: a cross-sectional study | 2015 | Samples 1 & 2 |
| Correlates of Total Sedentary Time and Screen Time in 9-11 Year-Old Children around the World: The International Study of Childhood Obesity, Lifestyle and the Environment | 2015 | Samples 1 & 2 |
| A Study of the Combined Effects of Physical Activity and Air Pollution on Mortality in Elderly Urban Residents: The Danish Diet, Cancer, and Health Cohort | 2015 | Samples 1 & 2 |
| Soil-Landscape Estimation and Evaluation Program (SLEEP) to predict spatial distribution of soil attributes for environmental modeling | 2015 | Samples 1 & 2 |
| Profiling Physical Activity, Diet, Screen and Sleep Habits in Portuguese Children | 2015 | Samples 1 & 2 |
| Energy Intake, Profile, and Dietary Sources in the Spanish Population: Findings of the ANIBES Study | 2015 | Samples 1 & 2 |
| Association of Markers of Inflammation with Sleep and Physical Activity Among People Living with HIV or AIDS | 2015 | Samples 1 & 2 |
| A novel safety assessment strategy applied to non-selective extracts | 2015 | Samples 1 & 2 |
| Sedentary Behavior Research Priorities-NHLBI/NIA Sedentary Behavior Workshop Summary | 2015 | Samples 1 & 2 |
| Age differences in the brain mechanisms of good taste | 2015 | Samples 1 & 2 |
| Exercise intensity prescription during heat stress: A brief review | 2015 | Samples 1 & 2 |
| Dietary and lifestyle patterns in relation to high blood pressure in children: the GRECO study | 2015 | Samples 1 & 2 |
| The Effect of Cardiorespiratory Fitness on Age-Related Lipids and Lipoproteins | 2015 | Samples 1 & 2 |
| Caffeine antiplasticization of amorphous poly(ethylene terephthalate): Effects on gas transport, thermal, and mechanical properties | 2015 | Samples 1 & 2 |
| Mediating role of television time, diet patterns, physical activity and sleep duration in the association between television in the bedroom and adiposity in 10 year-old children | 2015 | Samples 1 & 2 |
| Direct and self-reported measures of physical activity and sedentary behaviours by weight status in school-aged children: results from ISCOLE-Kenya | 2015 | Samples 1 & 2 |
| Evaluation of Drinks Contribution to Energy Intake in Summer and Winter | 2015 | Samples 1 & 2 |
| Multiple-Micronutrient Fortified Non-Dairy Beverage Interventions Reduce the Risk of Anemia and Iron Deficiency in School-Aged Children in Low-Middle Income Countries: A Systematic Review and Meta-Analysis ((i-iv)) | 2015 | Samples 1 & 2 |
| Identifying Children's Nocturnal Sleep Using 24-h Waist Accelerometry | 2015 | Samples 1 & 2 |
| Arterial blood pressure responses to short-term exposure to low and high traffic-related air pollution with and without moderate physical activity | 2015 | Samples 1 & 2 |
| Perceived Stress and ADHD Symptoms in Adults | 2015 | Samples 1 & 2 |
| A model for presenting accelerometer paradata in large studies: ISCOLE | 2015 | Samples 1 & 2 |
| Carbon Dioxide Sorption and Transport in Amorphous Poly(ethylene furanoate) | 2015 | Samples 1 & 2 |
| Effects of alcohol consumption and the metabolic syndrome on 10-year incidence of diabetes: The ATTICA study | 2015 | Samples 1 & 2 |
| Extremes of weight gain and weight loss with detailed assessments of energy balance: Illustrative case studies and clinical recommendations | 2015 | Samples 1 & 2 |
| Self-compassion as a moderator of thinness-related pressures' associations with thin-ideal internalization and disordered eating | 2015 | Samples 1 & 2 |
| Respiratory and inflammatory responses to short-term exposure to traffic-related air pollution with and without moderate physical activity | 2015 | Samples 1 & 2 |
| Objectively-measured sleep and its association with adiposity and physical activity in a sample of Canadian children | 2015 | Samples 1 & 2 |
| Toward precision irrigation for intensive strawberry cultivation | 2015 | Samples 1 & 2 |
| Exposure to 'healthy' fast food meal bundles in television advertisements promotes liking for fast food but not healthier choices in children | 2015 | Samples 1 & 2 |
| Correlates of objectively measured sedentary time and self-reported screen time in Canadian children | 2015 | Samples 1 & 2 |
| Cardiovascular risk factor distribution and subjective risk estimation in urban women - The BEFRI Study: a randomized cross-sectional study | 2015 | Samples 1 & 2 |
| Physical Aging in Amorphous Poly(ethylene furanoate): Enthalpic Recovery, Density, and Oxygen Transport Considerations | 2015 | Samples 1 & 2 |
| Intensity-Specific Leisure-Time Physical Activity and The Built Environment Among Brazilian Adults: A Best-Fit Model | 2015 | Samples 1 & 2 |
| Outcomes of the Rope Skipping 'STAR' Programme for Schoolchildren | 2015 | Samples 1 & 2 |
| The association between different types of exercise and energy expenditure in young nonoverweight and overweight adults | 2015 | Samples 1 & 2 |
| A Count Model to Study the Correlates of 60 Min of Daily Physical Activity in Portuguese Children | 2015 | Samples 1 & 2 |
| A Prospective Study of Fitness, Fatness, and Depressive Symptoms | 2015 | Samples 1 & 2 |
| Regional variation in temporal organization in American English | 2015 | Samples 1 & 2 |
| In vitro colonic catabolism of orange juice (poly)phenols | 2015 | Samples 1 & 2 |
| Shifting the Lens: Using Critical Race Theory and Latino Critical Theory to Re-Examine the History of School Desegregation | 2015 | Samples 1 & 2 |
| Improving wear time compliance with a 24-hour waist-worn accelerometer protocol in the International Study of Childhood Obesity, Lifestyle and the Environment (ISCOLE) | 2015 | Samples 1 & 2 |
| The ANIBES Study on Energy Balance in Spain: Design, Protocol and Methodology | 2015 | Samples 1 & 2 |
| Fructose Containing Sugars Do Not Raise Blood Pressure or Uric Acid at Normal Levels of Human Consumption | 2015 | Samples 1 & 2 |
| Ten-year (2002-2012) cardiovascular disease incidence and all-cause mortality, in urban Greek population: The ATTICA Study | 2015 | Samples 1 & 2 |
| Adherence to Mediterranean Diet Offers an Additive Protection Over the Use of Statin Therapy: Results from the ATTICA Study (2002-2012) | 2015 | Samples 1 & 2 |
| Understanding physical activity participation in spinal cord injured populations: Three narrative types for consideration | 2015 | Samples 1 & 2 |
| Macroinvertebrate assemblages of natural springs along an altitudinal gradient in the Bernese Alps, Switzerland | 2015 | Samples 1 & 2 |
| POLICY CHOICE, SOCIAL STRUCTURE, AND INTERNATIONAL TOURISM IN BUENOS AIRES, HAVANA, AND RIO DE JANEIRO | 2015 | Samples 1 & 2 |
| Newly derived children-based food index. An index that may detect childhood overweight and obesity | 2015 | Samples 1 & 2 |
| Utility of models of the gastrointestinal tract for assessment of the digestion and absorption of engineered nanomaterials released from food matrices | 2015 | Samples 1 & 2 |
| Resistant starch: a functional food that prevents DNA damage and chemical carcinogenesis | 2015 | Samples 1 & 2 |
| Foodservice employee substance abuse: is anyone getting the message? | 2015 | Samples 1 & 2 |
| OBESITY AND HEALTH-RELATED LIFESTYLE FACTORS IN THE GENERAL POPULATION IN ROMANIA: A CROSS SECTIONAL STUDY | 2015 | Samples 1 & 2 |
| Citrus limonin glucoside supplementation decreased biomarkers of liver disease and inflammation in overweight human adults | 2015 | Samples 1 & 2 |
| Water sorption in poly(ethylene furanoate) compared to poly(ethylene terephthalate). Part 1: Equilibrium sorption | 2014 | Samples 1 & 2 |
| Water sorption in poly(ethylene furanoate) compared to poly(ethylene terephthalate). Part 2: Kinetic sorption | 2014 | Samples 1 & 2 |
| Overcoming the challenges of conducting physical activity and built environment research in Latin America: IPEN Latin America | 2014 | Samples 1 & 2 |
| Neighborhood Environments and Objectively Measured Physical Activity in 11 Countries | 2014 | Samples 1 & 2 |
| The impact of social segregation on human mobility in developing and industrialized regions | 2014 | Samples 1 & 2 |
| A qualitative examination of the impact of microgrants to promote physical activity among adolescents | 2014 | Samples 1 & 2 |
| Physical Activity: Does Environment Make a Difference for Tension, Stress, Emotional Outlook, and Perceptions of Health Status? | 2014 | Samples 1 & 2 |
| Parent-Targeted Mobile Phone Intervention to Increase Physical Activity in Sedentary Children: Randomized Pilot Trial | 2014 | Samples 1 & 2 |
| Successful Scientist: What's the Winning Formula? | 2014 | Samples 1 & 2 |
| Overweight and Obesity in Portuguese Children: Prevalence and Correlates | 2014 | Samples 1 & 2 |
| Orange juice (poly)phenols are highly bioavailable in humans | 2014 | Samples 1 & 2 |
| Susceptibility artifacts detected on 3T MRI of the knee: frequency, change over time and associations with radiographic findings: data from the Joints on Glucosamine Study | 2014 | Samples 1 & 2 |
| Low/No Calorie Sweetened Beverage Consumption in the National Weight Control Registry | 2014 | Samples 1 & 2 |
| Hierarchical analysis of dietary, lifestyle and family environment risk factors for childhood obesity: the GRECO study | 2014 | Samples 1 & 2 |
| Applying real options to IT investment evaluation: The case of radio frequency identification (RFID) technology in the supply chain | 2014 | Samples 1 & 2 |
| Effects of Cardiorespiratory Fitness on Blood Pressure Trajectory With Aging in a Cohort of Healthy Men | 2014 | Samples 1 & 2 |
| Quantifying the benefits of vehicle pooling with shareability networks | 2014 | Samples 1 & 2 |
| Oxygen sorption and transport in amorphous poly(ethylene furanoate) | 2014 | Samples 1 & 2 |
| The Effect of Temperature and Menthol on Carbonation Bite | 2014 | Samples 1 & 2 |
| Report of an EU-US Symposium on Understanding Nutrition-Related Consumer Behavior: Strategies to Promote a Lifetime of Healthy Food Choices | 2014 | Samples 1 & 2 |
| Body adiposity index and incident hypertension: The Aerobics Center Longitudinal Study | 2014 | Samples 1 & 2 |
| Sweetened carbonated beverage consumption and cancer risk: meta-analysis and review | 2014 | Samples 1 & 2 |
| Correlates of sedentary time in children: a multilevel modelling approach | 2014 | Samples 1 & 2 |
| Structure, permeability, and rheology of supercritical CO2 dispersed polystyrene-clay nanocomposites | 2014 | Samples 1 & 2 |
| Leisure-Time Running Reduces All-Cause and Cardiovascular Mortality Risk | 2014 | Samples 1 & 2 |
| Individual, Family, and Community Predictors of Overweight and Obesity Among Colombian Children and Adolescents | 2014 | Samples 1 & 2 |
| Characteristics of the Built Environment in Relation to Objectively Measured Physical Activity Among Mexican Adults, 2011 | 2014 | Samples 1 & 2 |
| Independent and combined associations of total sedentary time and television viewing time with food intake patterns of 9-to 11-year-old Canadian children | 2014 | Samples 1 & 2 |
| Cost effectiveness of primary care referral to a commercial provider for weight loss treatment, relative to standard care: a modelled lifetime analysis | 2014 | Samples 1 & 2 |
| Cut Points of Muscle Strength Associated with Metabolic Syndrome in Men | 2014 | Samples 1 & 2 |
| Severity of injuries in different modes of transport, expressed with disability-adjusted life years (DALYs) | 2014 | Samples 1 & 2 |
| General optimization technique for high-quality community detection in complex networks | 2014 | Samples 1 & 2 |
| Perceived neighbourhood environmental attributes associated with adults' recreational walking: IPEN Adult study in 12 countries | 2014 | Samples 1 & 2 |
| Sucrose compared with artificial sweeteners: a clinical intervention study of effects on energy intake, appetite, and energy expenditure after 10 wk of supplementation in overweight subjects | 2014 | Samples 1 & 2 |
| Measurement Methods for the Oral Uptake of Engineered Nanomaterials from Human Dietary Sources: Summary and Outlook | 2014 | Samples 1 & 2 |
| Measurement Methods to Evaluate Engineered Nanomaterial Release from Food Contact Materials | 2014 | Samples 1 & 2 |
| Measurement Methods to Detect, Characterize, and Quantify Engineered Nanomaterials in Foods | 2014 | Samples 1 & 2 |
| Methods to Evaluate Uptake of Engineered Nanomaterials by the Alimentary Tract | 2014 | Samples 1 & 2 |
| Engineered Nanoscale Food Ingredients: Evaluation of Current Knowledge on Material Characteristics Relevant to Uptake from the Gastrointestinal Tract | 2014 | Samples 1 & 2 |
| Aerobic and Strength Training in Concomitant Metabolic Syndrome and Type 2 Diabetes | 2014 | Samples 1 & 2 |
| Exploring Universal Patterns in Human Home-Work Commuting from Mobile Phone Data | 2014 | Samples 1 & 2 |
| Longitudinal Algorithms to Estimate Cardiorespiratory Fitness Associations With Nonfatal Cardiovascular Disease and Disease-Specific Mortality | 2014 | Samples 1 & 2 |
| Objectively measured physical activity, sedentary time and sleep duration: independent and combined associations with adiposity in canadian children | 2014 | Samples 1 & 2 |
| Moderate Cardiorespiratory Fitness Is Positively Associated With Resting Metabolic Rate in Young Adults | 2014 | Samples 1 & 2 |
| A school-based rope skipping intervention for adolescents in Hong Kong: protocol of a matched-pair cluster randomized controlled trial | 2014 | Samples 1 & 2 |
| A cross-sectional examination of socio-demographic and school-level correlates of children's school travel mode in Ottawa, Canada | 2014 | Samples 1 & 2 |
| Correlates of objectively measured overweight/obesity and physical activity in Kenyan school children: results from ISCOLE-Kenya | 2014 | Samples 1 & 2 |
| Estimating human trajectories and hotspots through mobile phone data | 2014 | Samples 1 & 2 |
| Simulation of Population-Based Commuter Exposure to NO2 Using Different Air Pollution Models | 2014 | Samples 1 & 2 |
| Estimated Intakes and Sources of Total and Added Sugars in the Canadian Diet | 2014 | Samples 1 & 2 |
| Multinutrient-Fortified Juices Improve Vitamin D and Vitamin E Status in Children: A Randomized Controlled Trial | 2014 | Samples 1 & 2 |
| Low Fitness Partially Explains Resting Metabolic Rate Differences Between African American and White Women | 2014 | Samples 1 & 2 |
| Effect of Oral Glucosamine on Joint Structure in Individuals With Chronic Knee Pain | 2014 | Samples 1 & 2 |
| Kidney Disease: Improving Global Outcomes-an update | 2014 | Samples 1 & 2 |
| Measurement of Nanomaterials in Foods: Integrative Consideration of Challenges and Future Prospects | 2014 | Samples 1 & 2 |
| Dietary indices, cardiovascular risk factors and mortality in middle-aged adults: findings from the Aerobics Center Longitudinal Study | 2014 | Samples 1 & 2 |
| Dietary Sugar and Body Weight: Have We Reached a Crisis in the Epidemic of Obesity and Diabetes? | 2014 | Samples 1 & 2 |
| Computer Vision System Applied to Classification of "Manila" Mangoes During Ripening Process | 2014 | Samples 1 & 2 |
| In vitro metabolism of rebaudioside B, D, and M under anaerobic conditions: Comparison with rebaudioside A | 2014 | Samples 1 & 2 |
| Chain Mobility, Thermal, and Mechanical Properties of Poly(ethylene furanoate) Compared to Poly(ethylene terephthalate) | 2014 | Samples 1 & 2 |
| Consumption of Mixed Fruit-juice Drink and Vitamin C Reduces Postprandial Stress Induced by a High Fat Meal in Healthy Overweight Subjects | 2014 | Samples 1 & 2 |
| The Effect of the Timing of Meal Intake on Energy Metabolism during Moderate Exercise | 2014 | Samples 1 & 2 |
| Aromatics from biomass pyrolysis vapour using a bifunctional mesoporous catalyst | 2014 | Samples 1 & 2 |
| Resistance reminders: Dieters reduce energy intake after exposure to diet-congruent food images compared to control non-food images | 2014 | Samples 1 & 2 |
| Fructose vs. glucose and metabolism: do the metabolic differences matter? | 2014 | Samples 1 & 2 |
| Fruit juice drinks prevent endogenous antioxidant response to high-fat meal ingestion | 2014 | Samples 1 & 2 |
| Findings from an online behavioural weight management programme provided with or without a fortified diet beverage | 2014 | Samples 1 & 2 |
| A new insight into land use classification based on aggregated mobile phone data | 2014 | Samples 1 & 2 |
| Inflammation-mediating cytokine response to acute handcycling exercise with/without functional electrical stimulation-evoked lower-limb cycling | 2014 | Samples 1 & 2 |
| Description of the EUROBIS Program: A Combination of an Epode Community-Based and a Clinical Care Intervention to Improve the Lifestyles of Children and Adolescents with Overweight or Obesity | 2014 | Samples 1 & 2 |
| Obesity and Headache/Migraine: The Importance of Weight Reduction through Lifestyle Modifications | 2014 | Samples 1 & 2 |
| Entropy and the Predictability of Online Life | 2014 | Samples 1 & 2 |
| The Role of Glycemic Index and Glycemic Load In Cardiovascular Disease And Its Risk Factors: A Review of The Recent Literature | 2014 | Samples 1 & 2 |
| Enzymatic hydrolysis of hard-to-cook bean (Phaseolus vulgaris L.) protein concentrates and its effects on biological and functional properties | 2014 | Samples 1 & 2 |
| Effects of a multi-micronutrient-fortified beverage, with and without sugar, on growth and cognition in South African schoolchildren: a randomised, double-blind, controlled intervention | 2013 | Samples 1 & 2 |
| Delineating Geographical Regions with Networks of Human Interactions in an Extensive Set of Countries | 2013 | Samples 1 & 2 |
| Genetic predisposition to an adverse lipid profile limits the improvement in total cholesterol in response to weight loss | 2013 | Samples 1 & 2 |
| DETECTION OF Lactobacillus plantarum 299V USING MICROCANTILEVER-BASED BIOSENSOR WITH DYNAMIC FORCE MICROSCOPY | 2013 | Samples 1 & 2 |
| Slimming starters. Intake of a diet-congruent food reduces meal intake in active dieters | 2013 | Samples 1 & 2 |
| Maternal Inactivity: 45-Year Trends in Mothers' Use of Time | 2013 | Samples 1 & 2 |
| Validation of a Novel Protocol for Calculating Estimated Energy Requirements and Average Daily Physical Activity Ratio for the US Population: 2005-2006 | 2013 | Samples 1 & 2 |
| Evaluation of the mechanical damage on wheat starch granules by SEM, ESEM, AFM and texture image analysis | 2013 | Samples 1 & 2 |
| Chia (Salvia hispanica L.) seed mucilage release characterisation. A microstructural and image analysis study | 2013 | Samples 1 & 2 |
| A Prospective Study of Ideal Cardiovascular Health and Depressive Symptoms | 2013 | Samples 1 & 2 |
| Overstatement of Results in the Nutrition and Obesity Peer-Reviewed Literature | 2013 | Samples 1 & 2 |
| Health impact assessment of increasing public transport and cycling use in Barcelona: A morbidity and burden of disease approach | 2013 | Samples 1 & 2 |
| Identifying accelerometer nonwear and wear time in older adults | 2013 | Samples 1 & 2 |
| Prevalence of MRI-detected mediopatellar plica in subjects with knee pain and the association with MRI-detected patellofemoral cartilage damage and bone marrow lesions: data from the Joints On Glucosamine study | 2013 | Samples 1 & 2 |
| Validity of US Nutritional Surveillance: National Health and Nutrition Examination Survey Caloric Energy Intake Data, 1971-2010 | 2013 | Samples 1 & 2 |
| Commuter exposure to ultrafine particles in different urban locations, transportation modes and routes | 2013 | Samples 1 & 2 |
| The International Study of Childhood Obesity, Lifestyle and the Environment (ISCOLE): design and methods | 2013 | Samples 1 & 2 |
| Prospective study of alcohol consumption and the incidence of the metabolic syndrome in US men | 2013 | Samples 1 & 2 |
| Spinal Cord Injury Level and the Circulating Cytokine Response to Strenuous Exercise | 2013 | Samples 1 & 2 |
| The Energy Balance Study: The Design and Baseline Results for a Longitudinal Study of Energy Balance | 2013 | Samples 1 & 2 |
| Body Adiposity Index and All-Cause and Cardiovascular Disease Mortality in Men | 2013 | Samples 1 & 2 |
| Evidence mapping: methodologic foundations and application to intervention and observational research on sugar-sweetened beverages and health outcomes | 2013 | Samples 1 & 2 |
| Effect of Carbonation on Brain Processing of Sweet Stimuli in Humans | 2013 | Samples 1 & 2 |
| Proteomic analysis of the enzymes involved in the starch biosynthesis of maize with different endosperm type and characterization of the starch | 2013 | Samples 1 & 2 |
| Antioxidant activity of Vigna unguiculata L. walp and hard-to-cook Phaseolus vulgaris L. protein hydrolysates | 2013 | Samples 1 & 2 |
| Effect of the Great Activity Programme on healthy lifestyle behaviours in 7-11 year olds | 2013 | Samples 1 & 2 |
| Evaluation of seasonality on total water intake, water loss and water balance in the general population in Greece | 2013 | Samples 1 & 2 |
| Determinants of the Changes in Glycemic Control with Exercise Training in Type 2 Diabetes: A Randomized Trial | 2013 | Samples 1 & 2 |
| Dose Effect of Cardiorespiratory Exercise on Metabolic Syndrome in Postmenopausal Women | 2013 | Samples 1 & 2 |
| Bitterness values for traditional tonic plants of southern Africa | 2013 | Samples 1 & 2 |
| Comparison of Physical Activity Measures Using Mobile Phone-Based CalFit and Actigraph | 2013 | Samples 1 & 2 |
| Racial differences in the response of cardiorespiratory fitness to aerobic exercise training in Caucasian and African American postmenopausal women | 2013 | Samples 1 & 2 |
| Bioavailability of dietary (poly)phenols: a study with ileostomists to discriminate between absorption in small and large intestine | 2013 | Samples 1 & 2 |
| The association between breastfeeding, maternal smoking in utero, and birth weight with bone mass and fractures in adolescents: a 16-year longitudinal study | 2013 | Samples 1 & 2 |
| Improving estimates of air pollution exposure through ubiquitous sensing technologies | 2013 | Samples 1 & 2 |
| Construction and validation of a measure of integrative well-being in seven languages: The Pemberton Happiness Index | 2013 | Samples 1 & 2 |
| Sharing good NEWS across the world: developing comparable scores across 12 countries for the neighborhood environment walkability scale (NEWS) | 2013 | Samples 1 & 2 |
| Nine Months of Combined Training Improves Ex Vivo Skeletal Muscle Metabolism in Individuals With Type 2 Diabetes | 2013 | Samples 1 & 2 |
| Antioxidant and inflammatory response following high-fat meal consumption in overweight subjects | 2013 | Samples 1 & 2 |
| Inflammation and oxidative stress are lower in physically fit and active adults | 2013 | Samples 1 & 2 |
| Th17/Treg Imbalance in Murine Cystic Fibrosis Is Linked to Indoleamine 2,3-Dioxygenase Deficiency but Corrected by Kynurenines | 2013 | Samples 1 & 2 |
| Maximal Estimated Cardiorespiratory Fitness, Cardiometabolic Risk Factors, and Metabolic Syndrome in the Aerobics Center Longitudinal Study | 2013 | Samples 1 & 2 |
| Water flows, energy demand, and market analysis of the informal water sector in Kisumu, Kenya | 2013 | Samples 1 & 2 |
| Gustation assessment using the NIH Toolbox | 2013 | Samples 1 & 2 |
| 45-Year Trends in Women's Use of Time and Household Management Energy Expenditure | 2013 | Samples 1 & 2 |
| The intriguing metabolically healthy but obese phenotype: cardiovascular prognosis and role of fitness | 2013 | Samples 1 & 2 |
| Socio-economic and demographic determinants of childhood obesity prevalence in Greece: the GRECO (Greek Childhood Obesity) study | 2013 | Samples 1 & 2 |
| Beverage consumption habits "24/7" among British adults: association with total water intake and energy intake | 2013 | Samples 1 & 2 |
| Case-control and prospective studies of dietary alpha-linolenic acid intake and prostate cancer risk: a meta-analysis | 2013 | Samples 1 & 2 |
| The relationship of post-fire white ash cover to surface fuel consumption | 2013 | Samples 1 & 2 |
| ILSI Brazil International Workshop on Functional Foods: a narrative review of the scientific evidence in the area of carbohydrates, microbiome, and health | 2013 | Samples 1 & 2 |
| Steviol glycoside safety: Is the genotoxicity database sufficient? | 2013 | Samples 1 & 2 |
| Years of Life Gained Due to Leisure-Time Physical Activity in the U.S. | 2013 | Samples 1 & 2 |
| From model outputs to conservation action: Prioritizing locations for implementing agricultural best management practices in a Midwestern watershed | 2013 | Samples 1 & 2 |
| Risk of disordered eating attitudes among adolescents in seven Arab countries by gender and obesity: A cross-cultural study | 2013 | Samples 1 & 2 |
| Physical activity/fitness peaks during perimenopause and BMI change patterns are not associated with baseline activity/fitness in women: a longitudinal study with a median 7-year follow-up | 2013 | Samples 1 & 2 |
| Efficacy of a multi micronutrient-fortified drink in improving iron and micronutrient status among schoolchildren with low iron stores in India: a randomised, double-masked placebo-controlled trial | 2013 | Samples 1 & 2 |
| Exercise Training and Habitual Physical Activity A Randomized Controlled Trial | 2012 | Samples 1 & 2 |
| Relationship between Use of Water from Community-Scale Water Treatment Refill Kiosks and Childhood Diarrhea in Jakarta | 2012 | Samples 1 & 2 |
| Beverages containing soluble fiber, caffeine, and green tea catechins suppress hunger and lead to less energy consumption at the next meal | 2012 | Samples 1 & 2 |
| Hydration profile and influence of beverage contents on fluid intake by women during outdoor recreational walking | 2012 | Samples 1 & 2 |
| Effect of Legumes as Part of a Low Glycemic Index Diet on Glycemic Control and Cardiovascular Risk Factors in Type 2 Diabetes Mellitus A Randomized Controlled Trial | 2012 | Samples 1 & 2 |
| Replacing car trips by increasing bike and public transport in the greater Barcelona metropolitan area: A health impact assessment study | 2012 | Samples 1 & 2 |
| Effects of Muscular Strength on Cardiovascular Risk Factors and Prognosis | 2012 | Samples 1 & 2 |
| Longitudinal Cardiorespiratory Fitness Algorithms for Clinical Settings | 2012 | Samples 1 & 2 |
| Thirst and hydration status in everyday life | 2012 | Samples 1 & 2 |
| Identification of hemostatic genes expressed in human and rat leg muscles and a novel gene (LPP1/PAP2A) suppressed during prolonged physical inactivity (sitting) | 2012 | Samples 1 & 2 |
| Inappropriate Fiddling with Statistical Analyses to Obtain a Desirable P-value: Tests to Detect its Presence in Published Literature | 2012 | Samples 1 & 2 |
| Evidence that women meeting physical activity guidelines do not sit less: An observational inclinometry study | 2012 | Samples 1 & 2 |
| Ideal Cardiovascular Health and Mortality: Aerobics Center Longitudinal Study | 2012 | Samples 1 & 2 |
| Vitamin D deficiency in Tasmania: a whole of life perspective | 2012 | Samples 1 & 2 |
| Willingness to Pay for Obesity Pharmacotherapy | 2012 | Samples 1 & 2 |
| Effects of cardiorespiratory fitness on aging: glucose trajectory in a cohort of healthy men | 2012 | Samples 1 & 2 |
| Theanine extends lifespan of adult Caenorhabditis elegans | 2012 | Samples 1 & 2 |
| The Effect of Exercise Training Modality on Serum Brain Derived Neurotrophic Factor Levels in Individuals with Type 2 Diabetes | 2012 | Samples 1 & 2 |
| OPTICAL, MICROSTRUCTURAL, FUNCTIONAL AND NANOMECHANICAL PROPERTIES OF Aloe vera GEL/GELLAN GUM EDIBLE FILMS | 2012 | Samples 1 & 2 |
| The effect of different doses of aerobic exercise training on endothelial function in postmenopausal women with elevated blood pressure: results from the DREW study | 2012 | Samples 1 & 2 |
| An Economic Analysis of Traditional and Technology-Based Approaches to Weight Loss | 2012 | Samples 1 & 2 |
| Gelation and microstructure of dilute gellan solutions with calcium ions | 2012 | Samples 1 & 2 |
| The implications of megatrends in information and communication technology and transportation for changes in global physical activity | 2012 | Samples 1 & 2 |
| The effect of a healthy lifestyle programme on 8-9 year olds from social disadvantage | 2012 | Samples 1 & 2 |
| Exercise Frequency Is Related to Psychopathology but Not Neurocognitive Function | 2012 | Samples 1 & 2 |
| Annual survival and breeding dispersal of a seabird adapted to a stable environment: implications for conservation | 2012 | Samples 1 & 2 |
| A-Z of nutritional supplements: dietary supplements, sports nutrition foods and ergogenic aids for health and performance-Part 33 | 2012 | Samples 1 & 2 |
| Cardiorespiratory Fitness Reduces the Risk of Incident Hypertension Associated With a Parental History of Hypertension | 2012 | Samples 1 & 2 |
| Attention Deficit Hyperactivity Disorder Subtypes and Their Relation to Cognitive Functioning, Mood States, and Combat Stress Symptomatology in Deploying US Soldiers | 2012 | Samples 1 & 2 |
| Risk factors for magnetic resonance imaging-detected patellofemoral and tibiofemoral cartilage loss during a six-month period: The Joints On Glucosamine study | 2012 | Samples 1 & 2 |
| Evaluating the concerns of pregnant women with epilepsy: A focus group approach | 2012 | Samples 1 & 2 |
| A global Water Quality Index and hot-deck imputation of missing data | 2012 | Samples 1 & 2 |
| The Obesity Paradox, Cardiorespiratory Fitness, and Coronary Heart Disease | 2012 | Samples 1 & 2 |
| The effect of different doses of aerobic exercise training on exercise blood pressure in overweight and obese postmenopausal women | 2012 | Samples 1 & 2 |
| Analyses of single nucleotide polymorphisms in selected nutrient-sensitive genes in weight-regain prevention: the DIOGENES study | 2012 | Samples 1 & 2 |
| The Effects of Fructose Intake on Serum Uric Acid Vary among Controlled Dietary Trials | 2012 | Samples 1 & 2 |
| Hydration and cognitive performance | 2012 | Samples 1 & 2 |
| Cardiovascular Drift During Heat Stress: Implications for Exercise Prescription | 2012 | Samples 1 & 2 |
| Effect of Different Doses of Aerobic Exercise Training on Total Bilirubin Levels | 2012 | Samples 1 & 2 |
| Effect of Fructose on Blood Pressure A Systematic Review and Meta-Analysis of Controlled Feeding Trials | 2012 | Samples 1 & 2 |
| Trial of Prevention and Reduction of Obesity Through Active Living in Clinical Settings A Randomized Controlled Trial | 2012 | Samples 1 & 2 |
| A Genome-Wide Association Study Identifies rs2000999 as a Strong Genetic Determinant of Circulating Haptoglobin Levels | 2012 | Samples 1 & 2 |
| CHANGING PREDICTORS OF SPATIAL AND TEMPORAL VARIABILITY IN STOCKING RATES IN A SEVERELY DEGRADED COMMUNAL RANGELAND | 2012 | Samples 1 & 2 |
| Associations of cardiorespiratory fitness and parental history of diabetes with risk of type 2 diabetes | 2012 | Samples 1 & 2 |
| The water balance questionnaire: design, reliability and validity of a questionnaire to evaluate water balance in the general population | 2012 | Samples 1 & 2 |
| Changes in Fitness and Fatness on the Development of Cardiovascular Disease Risk Factors Hypertension, Metabolic Syndrome, and Hypercholesterolemia | 2012 | Samples 1 & 2 |
| Metabolic syndrome in a Mediterranean pediatric cohort: prevalence using International Diabetes Federation-derived criteria and associations with adiponectin and leptin | 2012 | Samples 1 & 2 |
| Association between Leisure Time Physical Activity and Depressive Symptoms in Men | 2012 | Samples 1 & 2 |
| Effect of Dilute CHO Beverages on Performance in Cool and Warm Environments | 2012 | Samples 1 & 2 |
| Non-toxic poly(ethylene terephthalate)/clay nanocomposites with enhanced barrier properties | 2012 | Samples 1 & 2 |
| Chain Dynamics in Antiplasticized and Annealed Poly(ethylene terephthalate) Determined by Solid-State NMR and Correlated with Enhanced Barrier Properties | 2012 | Samples 1 & 2 |
| Antiplasticization-based enhancement of poly(ethylene terephthalate) barrier properties | 2012 | Samples 1 & 2 |
| Inflammation in aspergillosis: the good, the bad, and the therapeutic | 2012 | Samples 1 & 2 |
| Combined Fruit and Vegetable Intake Is Correlated with Improved Inflammatory and Oxidant Status from a Cross-Sectional Study in a Community Setting | 2012 | Samples 1 & 2 |
| High Fat Meal Increase of IL-17 is Prevented by Ingestion of Fruit Juice Drink in Healthy Overweight Subjects | 2012 | Samples 1 & 2 |
| Effects of Cooking Temperatures on the Physicochemical Properties and Consumer Acceptance of Chicken Stock | 2012 | Samples 1 & 2 |
| Calcium and vitamin D supplementation is associated with decreased abdominal visceral adipose tissue in overweight and obese adults | 2012 | Samples 1 & 2 |
| Long-Term Effects of Changes in Cardiorespiratory Fitness and Body Mass Index on All-Cause and Cardiovascular Disease Mortality in Men The Aerobics Center Longitudinal Study | 2011 | Samples 1 & 2 |
| Usefulness of Serum Bilirubin and Cardiorespiratory Fitness as Predictors of Mortality in Men | 2011 | Samples 1 & 2 |
| Quality of reviews on sugar-sweetened beverages and health outcomes: a systematic review | 2011 | Samples 1 & 2 |
| A Prospective Study of Fasting Plasma Glucose and Risk of Stroke in Asymptomatic Men | 2011 | Samples 1 & 2 |
| Self-rated health status and cardiorespiratory fitness as predictors of mortality in men | 2011 | Samples 1 & 2 |
| Primary care referral to a commercial provider for weight loss treatment versus standard care: a randomised controlled trial | 2011 | Samples 1 & 2 |
| The role of a pre-load beverage on gastric volume and food intake: comparison between non-caloric carbonated and non-carbonated beverage | 2011 | Samples 1 & 2 |
| Heart rate recovery after treadmill exercise testing is an independent predictor of stroke incidence in men with metabolic syndrome | 2011 | Samples 1 & 2 |
| Clinical utility of inflammatory markers and advanced lipoprotein testing: Advice from an expert panel of lipid specialists | 2011 | Samples 1 & 2 |
| Influence of cardiac rehabilitation on natriuretic peptides | 2011 | Samples 1 & 2 |
| Identification of a Novel Effector Domain of BIN1 for Cancer Suppression | 2011 | Samples 1 & 2 |
| Combined Impact of Lifestyle Factors on Cancer Mortality in Men | 2011 | Samples 1 & 2 |
| Semiquantitative assessment of subchondral bone marrow edema-like lesions and subchondral cysts of the knee at 3T MRI: A comparison between intermediate-weighted fat-suppressed spin echo and Dual Echo Steady State sequences | 2011 | Samples 1 & 2 |
| Immature survival and age at first breeding of Damara Terns: conservation from a non-breeding perspective | 2011 | Samples 1 & 2 |
| Upper respiratory tract infection is reduced in physically fit and active adults | 2011 | Samples 1 & 2 |
| The health risks and benefits of cycling in urban environments compared with car use: health impact assessment study | 2011 | Samples 1 & 2 |
| Edmonton Obesity Staging System: association with weight history and mortality risk | 2011 | Samples 1 & 2 |
| Very high childhood obesity prevalence and low adherence rates to the Mediterranean diet in Greek children: The GRECO study | 2011 | Samples 1 & 2 |
| How many steps/day are enough? For older adults and special populations | 2011 | Samples 1 & 2 |
| How Many Steps/Day are Enough? for Children and Adolescents | 2011 | Samples 1 & 2 |
| Physical activity and body composition outcomes of the GreatFun2Run intervention at 20 month follow-up | 2011 | Samples 1 & 2 |
| Genetic variation within IL18 is associated with insulin levels, insulin resistance and postprandial measures | 2011 | Samples 1 & 2 |
| Does a short breastfeeding period protect from FTO-induced adiposity in children? | 2011 | Samples 1 & 2 |
| Quercetin with vitamin C and niacin does not affect body mass or composition | 2011 | Samples 1 & 2 |
| A Research Model for Investigating the Effects of Artificial Food Colorings on Children With ADHD | 2011 | Samples 1 & 2 |
| Green Tea Polyphenols Provide Photoprotection, Increase Microcirculation, and Modulate Skin Properties of Women | 2011 | Samples 1 & 2 |
| High sodium intake of children through 'hidden' food sources and its association with the Mediterranean diet: the GRECO study | 2011 | Samples 1 & 2 |
| Electronic feedback in a diet- and physical activity-based lifestyle intervention for weight loss: a randomized controlled trial | 2011 | Samples 1 & 2 |
| A Prospective Study of Muscular Strength and All-Cause Mortality in Men With Hypertension | 2011 | Samples 1 & 2 |
| Independent and Joint Associations of Physical Activity and Fitness on Stroke in Men | 2011 | Samples 1 & 2 |
| Improving health through policies that promote active travel: A review of evidence to support integrated health impact assessment | 2011 | Samples 1 & 2 |
| Low-pH Cola Beverages Do Not Affect Women's Iron Absorption from a Vegetarian Meal | 2011 | Samples 1 & 2 |
| Comparisons of leisure-time physical activity and cardiorespiratory fitness as predictors of all-cause mortality in men and women | 2011 | Samples 1 & 2 |
| Concordance of freshwater and terrestrial biodiversity | 2011 | Samples 1 & 2 |
| Influence of Quercetin Supplementation on Disease Risk Factors in Community-Dwelling Adults | 2011 | Samples 1 & 2 |
| An age-dependent diet-modified effect of the PPAR gamma Pro12Ala polymorphism in children | 2011 | Samples 1 & 2 |
| From an Inactive and Obese to a Fit Child: How Long Is the Way? Czech Experiences | 2011 | Samples 1 & 2 |
| A Review of the Literature on Policies Directed at the Youth Consumption of Sugar Sweetened Beverages | 2011 | Samples 1 & 2 |
| A Narrative Review of Physical Activity, Nutrition, and Obesity to Cognition and Scholastic Performance across the Human Lifespan | 2011 | Samples 1 & 2 |
| Dietary Education in School-Based Childhood Obesity Prevention Programs | 2011 | Samples 1 & 2 |
| Bioavailability of Iron and Zinc from Multiple Micronutrient Fortified Beverage Premixes in Caco-2 Cell Model | 2011 | Samples 1 & 2 |
| The relation of low glycaemic index fruit consumption to glycaemic control and risk factors for coronary heart disease in type 2 diabetes | 2011 | Samples 1 & 2 |
| In Fitness and Health? A Prospective Study of Changes in Marital Status and Fitness in Men and Women | 2011 | Samples 1 & 2 |
| Cardiorespiratory fitness and risk of prostate cancer: Findings from the Aerobics Center Longitudinal Study | 2011 | Samples 1 & 2 |
| Does Caffeine Added to Carbohydrate Provide Additional Ergogenic Benefit for Endurance? | 2011 | Samples 1 & 2 |
| Electrochemically activated water as an alternative to chlorine for decentralized disinfection | 2011 | Samples 1 & 2 |
| Fortified juice drink improved iron and zinc status of schoolchildren | 2011 | Samples 1 & 2 |
| Acceleration Tolerance After Ingestion of a Commercial Energy Drink | 2010 | Samples 1 & 2 |
| Diets with High or Low Protein Content and Glycemic Index for Weight-Loss Maintenance. | 2010 | Samples 1 & 2 |
| Effects of Aerobic and Resistance Training on Hemoglobin A(1c) Levels in Patients With Type 2 Diabetes A Randomized Controlled Trial | 2010 | Samples 1 & 2 |
| Psychological Well-Being, Cardiorespiratory Fitness, and Long-Term Survival | 2010 | Samples 1 & 2 |
| Vitamin D content and variability in fluid milks from a US Department of Agriculture nationwide sampling to update values in the National Nutrient Database for Standard Reference | 2010 | Samples 1 & 2 |
| A Low-Calorie Beverage Supplemented with Low-Viscosity Pectin Reduces Energy Intake at a Subsequent Meal | 2010 | Samples 1 & 2 |
| Consumption of Vegetables, Cooked Meals, and Eating Dinner is Negatively Associated with Overweight Status in Children | 2010 | Samples 1 & 2 |
| Mortality trends in the general population: the importance of cardiorespiratory fitness | 2010 | Samples 1 & 2 |
| Evaluation of concentration-response options for diacetyl in support of occupational risk assessment | 2010 | Samples 1 & 2 |
| Muscular Strength Is Inversely Related to Prevalence and Incidence of Obesity in Adult Men | 2010 | Samples 1 & 2 |
| Development of hydration strategies to optimize performance for athletes in high-intensity sports and in sports with repeated intense efforts | 2010 | Samples 1 & 2 |
| A 12-week supplementation with quercetin does not affect natural killer cell activity, granulocyte oxidative burst activity or granulocyte phagocytosis in female human subjects | 2010 | Samples 1 & 2 |
| Exposure of Africa's freshwater biodiversity to a changing climate | 2010 | Samples 1 & 2 |
| Effect of Positive Health Factors and All-Cause Mortality in Men | 2010 | Samples 1 & 2 |
| Quercetin supplementation and upper respiratory tract infection: A randomized community clinical trial | 2010 | Samples 1 & 2 |
| Metabolic syndrome and risk of death from cancers of the digestive system | 2010 | Samples 1 & 2 |
| Effects of casting and post casting annealing on xylene isomer transport properties of Torlon (R) 4000T films | 2010 | Samples 1 & 2 |
| The impact of combined health factors on cardiovascular disease mortality | 2010 | Samples 1 & 2 |
| Falls Among Adults The Association of Cardiorespiratory Fitness and Physical Activity with Walking-Related Falls | 2010 | Samples 1 & 2 |
| Clarification of pineapple juice by microfiltration | 2010 | Samples 1 & 2 |
| The variable plasma quercetin response to 12-week quercetin supplementation in humans | 2010 | Samples 1 & 2 |
| Bioavailability of multiple components following acute ingestion of a polyphenol-rich juice drink | 2010 | Samples 1 & 2 |
| Fortification of orange juice with vitamin D-2 or vitamin D-3 is as effective as an oral supplement in maintaining vitamin D status in adults | 2010 | Samples 1 & 2 |
| Phenotypic and genotypic characteristics of Lactococcus lactis strains isolated from different ecosystems | 2010 | Samples 1 & 2 |
| Snacking patterns according to location among Northern Ireland children | 2010 | Samples 1 & 2 |
| Influence of Cardiorespiratory Fitness on Lung Cancer Mortality | 2010 | Samples 1 & 2 |
| Sedentary Behaviors Increase Risk of Cardiovascular Disease Mortality in Men | 2010 | Samples 1 & 2 |
| Epoch Length and Its Effect on Physical Activity Intensity | 2010 | Samples 1 & 2 |
| Variants in ADCY5 and near CCNL1 are associated with fetal growth and birth weight | 2010 | Samples 1 & 2 |
| Reliability and Validity of a School Recess Physical Activity Recall in Spanish Youth | 2010 | Samples 1 & 2 |
| A study of fluid intake from beverages in a sample of healthy French children, adolescents and adults | 2010 | Samples 1 & 2 |
| Effect of Various Carbohydrate-Electrolyte Fluids on Cycling Performance and Maximal Voluntary Contraction | 2010 | Samples 1 & 2 |
| Systematic review: the effects of carbonated beverages on gastro-oesophageal reflux disease | 2010 | Samples 1 & 2 |
| Antiplasticization and plasticization of Matrimid (R) asymmetric hollow fiber membranes-Part A. Experimental | 2010 | Samples 1 & 2 |
| Antiplasticization and plasticization of Matrimid (R) asymmetric hollow fiber membranes. Part B. Modeling | 2010 | Samples 1 & 2 |
| Institutional support and in situ conservation in Mexico: biases against small-scale maize farmers in post-NAFTA agricultural policy | 2010 | Samples 1 & 2 |
| Physical activity, cardiorespiratory fitness and the incidence of type 2 diabetes in a prospective study of men | 2010 | Samples 1 & 2 |
| Naphthyl methacrylate-based monolithic column for RP-CEC via hydrophobic and pi interactions | 2010 | Samples 1 & 2 |
| Dairy intake associates with the IGF rs680 polymorphism to height variation in periadolescent children | 2010 | Samples 1 & 2 |
| Identification of Metabolites in Human Plasma and Urine after Consumption of a Polyphenol-Rich Juice Drink | 2010 | Samples 1 & 2 |
| Accumulation of (H2O)-H-2 in plasma and eccrine sweat during exercise-heat stress | 2010 | Samples 1 & 2 |
| Quercetin supplementation does not alter antioxidant status in humans | 2010 | Samples 1 & 2 |
| KDIGO clinical practice guideline for the care of kidney transplant recipients: a summary | 2010 | Samples 1 & 2 |
| Muscular Strength and Incident Hypertension in Normotensive and Prehypertensive Men | 2010 | Samples 1 & 2 |
| Quercetin's Influence on Exercise Performance and Muscle Mitochondrial Biogenesis | 2010 | Samples 1 & 2 |
| Depressive symptoms predict exaggerated inflammatory responses to an in vivo immune challenge among pregnant women | 2010 | Samples 1 & 2 |
| APOE, CETP and LPL genes show strong association with lipid levels in Greek children | 2010 | Samples 1 & 2 |
| ADIPOQ gene polymorphism rs1501299 interacts with fibre intake to affect adiponectin concentration in children: the GENe-Diet Attica Investigation on childhood obesity | 2009 | Samples 1 & 2 |
| Toluene and n-heptane sorption in Matrimid (R) asymmetric hollow fiber membranes | 2009 | Samples 1 & 2 |
| Heterogeneous Effects of Fructose on Blood Lipids in Individuals With Type 2 Diabetes Systematic review and meta-analysis of experimental trials in humans | 2009 | Samples 1 & 2 |
| Dietary quercetin supplementation is not ergogenic in untrained men | 2009 | Samples 1 & 2 |
| Multicolumn Separation Platform for Simultaneous Depletion and Prefractionation Prior to 2-DE for Facilitating In-Depth Serum Proteomics Profiling | 2009 | Samples 1 & 2 |
| Peroxisome proliferator-activated receptor-gamma (PPAR gamma) Pro12Ala polymorphism and risk for pediatric obesity | 2009 | Samples 1 & 2 |
| Pomegranate Fruit Extract Impairs Invasion and Motility in Human Breast Cancer | 2009 | Samples 1 & 2 |
| Sorption of lower alcohols in poly(ethylene terephthalate) | 2009 | Samples 1 & 2 |
| Funding food science and nutrition research: financial conflicts and scientific integrity | 2009 | Samples 1 & 2 |
| Tracking of bone mass from childhood to adolescence and factors that predict deviation from tracking | 2009 | Samples 1 & 2 |
| Monolithic silica capillary columns having immobilized lectins and surface bound polar functionalities for lectin affinity and normal phase nano-LC and CEC of glycoconjugates, respectively | 2009 | Samples 1 & 2 |
| Docosahexaenoic Acid (DHA) Supplementation of Orange Juice Increases Plasma Phospholipid DHA Content of Children | 2009 | Samples 1 & 2 |
| Comparison of Analysis of Vitamin D-3 in Foods Using Ultraviolet and Mass Spectrometric Detection | 2009 | Samples 1 & 2 |
| Relative bioavailability of micronized, dispersible ferric pyrophosphate added to an apple juice drink | 2009 | Samples 1 & 2 |
| Prolonged insula activation during perception of aftertaste | 2009 | Samples 1 & 2 |
| Effect of a school-based intervention to promote healthy lifestyles in 7-11 year old children | 2009 | Samples 1 & 2 |
| Sorption and transport of methanol in poly(ethylene terephthalate) | 2009 | Samples 1 & 2 |
| The Key Events Dose-Response Framework: A Cross-Disciplinary Mode-of-Action Based Approach to Examining Dose-Response and Thresholds | 2009 | Samples 1 & 2 |
| Application of Key Events Analysis to Chemical Carcinogens and Noncarcinogens | 2009 | Samples 1 & 2 |
| Application of a Key Events Dose-Response Analysis to Nutrients: A Case Study with Vitamin A (Retinol) | 2009 | Samples 1 & 2 |
| The Key Events Dose-Response Framework: Its Potential for Application to Foodborne Pathogenic Microorganisms | 2009 | Samples 1 & 2 |
| The Key Events Dose-Response Framework: A Foundation for Examining Variability in Elicitation Thresholds for Food Allergens | 2009 | Samples 1 & 2 |
| Liquid-phase-based separation systems for depletion, prefractionation and enrichment of proteins in biological fluids for in-depth proteomics analysis | 2009 | Samples 1 & 2 |
| Neutral polar methacrylate-based monoliths for normal phase nano-LC and CEC of polar species including N-glycans | 2009 | Samples 1 & 2 |
| Is soft drink consumption associated with body composition? A cross-sectional study in Spanish adolescents | 2009 | Samples 1 & 2 |
| Assessing vitamin D contents in foods and supplements: challenges and needs | 2008 | Samples 1 & 2 |
| Analyzing vitamin D in foods and supplements: methodologic challenges | 2008 | Samples 1 & 2 |
| The impact of a school-based safe water and hygiene programme on knowledge and practices of students and their parents: Nyanza Province, western Kenya, 2006 | 2008 | Samples 1 & 2 |

Source: Compiled by the authors using data from Web of Science.

**R function used to scrape data from Web of Science**

###########################################################################

# R function to retrieve data from a Web of Science search #

# The function takes on two arguments: #

# (1) the URL from the first resulting article from the search; #

# (2) the total number of results in the search; #

###########################################################################

swoc <- function(x,k){

### Packages

require("rvest")

require("curl")

require("plyr")

### Trim leading function

trim.leading <- function (x) sub("^\\s+", "", x)

trim <- function (x) gsub("^\\s+|\\s+$", "", x)

### Arguments

res.length <- k

### ###

### Save webpages

print(paste("Downloading", res.length, "html files (one for each article entry in WoS)..."))

for(i in 1:res.length){

ptm <- proc.time()

print(paste(i, "/", k))

u <- paste(substr(x, 1, nchar(x)-1),i,sep='')

webpage <- curl_download(u, paste0("article", i, ".html"), handle=new_handle())

Sys.sleep(2)

time <- proc.time() - ptm

print(paste("Finished in", round(time[3]), "seconds"))

}

### ###

### Loop through saved files

ws.df <- NULL

print(paste("Looping through", res.length, "html files, scraping content and building final data frame..."))

for (i in 1:res.length){

ptm <- proc.time()

print(paste(i, "/", k))

final.data <- data.frame(page = i, author=NA, title=NA, journal=NA, year=NA, funding_agency = NA, cited=NA,

keywords = NA, grant_number = NA, funding_text=NA, woscat = NA, abstract=NA)

ws.page <- read_html(paste0("article", i, ".html"))

## Information blocks

page <- as.matrix(as.list(ws.page %>% html_nodes(".FR_field") %>% html_text()))

rownames(page) <- apply(page, 1 , function(x) gsub("(.*\n)(.*)(:.*)" ,"\\2", x))

rownames(page) <- noquote(rownames(page))

block <- as.matrix(as.list(ws.page %>% html_nodes(".block-record-info") %>% html_text()))

rownames(block) <- apply(block, 1 , function(x) gsub("(.*\n)(.*)(\n\n\n.*)" ,"\\2", x))

### Build data frames

if (length(gsub('.*:(.*)','\\1',page[which(rownames(page) == "Web of Science Categories"),]) > 0)){

final.data$woscat <- gsub('.*:(.*)','\\1',page[which(rownames(page) == "Web of Science Categories"),])}

if (length(gsub(c("\n"), "",gsub(c("\240"), "",gsub(c("\302"), "", gsub('.*text(.*)','\\1',block[grep("Funding Agency", block),]))))) > 0){

final.data$funding_text <- gsub(c("\n"), "",gsub(c("\240"), "",gsub(c("\302"), "", gsub('.*text(.*)','\\1',block[grep("Funding Agency", block),]))))

}else{final.data$funding_text <- NA}

final.data$journal <- gsub("(.*\n)(.*)(\n.*)", "\\2" ,((ws.page %>% html_nodes(".sourceTitle") %>% html_text())[1]))

final.data$institutions <- paste(unique(ws.page %>% html_nodes("preferred_org") %>% html_text()), collapse=",")

final.data$title <- gsub("(.*\n)(.*)(\n.*)", "\\2" ,(ws.page %>% html_nodes(".title") %>% html_text()))

final.data$year <- gsub("(.*:\n)(.*)(\n.*)" ,"\\2", page[which(rownames(page)=="Published"),])

final.data$author <- gsub("(.*\nBy:)(.*)(\n.*)" ,"\\2", page[which(rownames(page)=="By"),])

final.data$author <- gsub("(?:\\(.*?\\)|\\.)(*SKIP)(*F)|[\\w' ,\\\"]+", " ", final.data$author, perl=TRUE) # remove text outside parentheses

final.data$author <- gsub("\\)\\[\n \\]\n ",'', final.data$author, perl=TRUE)

final.data$author <- gsub(" -", "", gsub("\\(",'', gsub("\n :","", gsub("\\)", "", final.data$author))))

final.data$author <- trim.leading(final.data$author)

if (length(gsub("(.*:\n)(.*)(\n.*)" ,"\\2", page[which(rownames(page)=="Times Cited in Web of Science Core Collection"),])) > 0){

final.data$cited <- as.numeric(gsub("(.*:\n)(.*)(\n.*)" ,"\\2", page[which(rownames(page)=="Times Cited in Web of Science Core Collection"),]))}

if(length(which(rownames(page)=="KeyWords Plus")) > 0) {

final.data$keywords <- tolower(gsub("(.*:)(.*)(\n.*)" ,"\\2", page[which(rownames(page)=="KeyWords Plus"),]))

}

if(length(which(rownames(page)=="Author Keywords")) > 0) {

final.data$keywords <- tolower(gsub("(.*:)(.*)(\n.*)" ,"\\2", page[which(rownames(page)=="Author Keywords"),]))

}

if(length(gsub('(.*)(\\(C\\).*)', '\\1', gsub(c("\n"), "", gsub(c("\nAbstract"), "", gsub('.*text(.*)','\\1',block[grep("Abstract", block),]))))) > 0){

final.data$abstract <- gsub('(.*)(\\(C\\).*)', '\\1', gsub(c("\n"), "", gsub(c("\nAbstract"), "", gsub('.*text(.*)','\\1',block[grep("Abstract", block),]))))

} else {final.data$abstract <- NA}

funding.info <- as.list(gsub("\240", "", gsub("\302", "", ws.page %>% html_nodes(".FR_table_borders") %>% html_nodes(".fr_data_row") %>% html_text())))

## Funding information

if(length(funding.info) > 0){

final.data <- final.data[rep(seq_len(nrow(final.data)), length(funding.info)), ]

dat <- data.frame(funding_agency = rep(NA, length(funding.info)), grant_number= rep(NA, length(funding.info)))

for (j in 1:length(funding.info)){

fa <- as.matrix(unlist(strsplit(gsub("\n\n", "", funding.info[j]),"\n")))

fa <- as.matrix(fa[-which(fa==""),])

if(nrow(fa)== 1){dat$funding_agency[j] <- fa[1]

} else {

dat$funding_agency[j] <- fa[1]

dat$grant_number[j] <- fa[2]

}

}

final.data$funding_agency <- dat$funding_agency

final.data$grant_number <- dat$grant_number

}

ws.df <- rbind(ws.df, final.data)

time <- proc.time() - ptm

print(paste("Finished in", round(time[3]), "seconds"))

}

return(ws.df)

}

## Worked Example ##

## Set parameters

url <- "https://apps.webofknowledge.com/full_record.do?product=WOS&search_mode=AdvancedSearch&qid=1&SID=Y15nyBc71mXqBiA2M7h&page=1&doc=1"

k <- 779

## Retrieve data

wos.search <- swoc(url,k)

rm(list=setdiff(ls(),"wos.search"))
